# Adiponectin and Asthma: Knowns, Unknowns and Controversies

**DOI:** 10.3390/ijms22168971

**Published:** 2021-08-20

**Authors:** Marina Ruxandra Otelea, Oana Cristina Arghir, Corina Zugravu, Agripina Rascu

**Affiliations:** 1 Clinical Department 5, Faculty of General medicine, University of Medicine and Pharmacy Carol Davila, 020021 Bucharest, Romania; agripina.rascu@umfcd.ro; 2 Department of Pneumology, Faculty of Medicine, Ovidius University of Constanta, 900470 Constanta, Romania; Toana.arghir@univ-ovidius.ro; 3 National Institute of Public Health, 050463 Bucharest, Romania; corina.zugravu@umfcd.ro; 4 Department of Hygiene and Ecology, Faculty of Nursing and Midwifery, University of Medicine and Pharmacy Carol Davila, 020021 Bucharest, Romania; 5 Department of Occupational Medicine, Colentina Clinical Hospital, 020125 Bucharest, Romania

**Keywords:** adiponectin, asthma, obesity, eosinophilic inflammation, neutrophilic inflammation, macrophages

## Abstract

Adiponectin is an adipokine associated with the healthy obese phenotype. Adiponectin increases insulin sensitivity and has cardio and vascular protection actions. Studies related to adiponectin, a modulator of the innate and acquired immunity response, have suggested a role of this molecule in asthma. Studies based on various asthma animal models and on the key cells involved in the allergic response have provided important insights about this relation. Some of them indicated protection and others reversed the balance towards negative effects. Many of them described the cellular pathways activated by adiponectin, which are potentially beneficial for asthma prevention or for reduction in the risk of exacerbations. However, conclusive proofs about their efficiency still need to be provided. In this article, we will, briefly, present the general actions of adiponectin and the epidemiological studies supporting the relation with asthma. The main focus of the current review is on the mechanisms of adiponectin and the impact on the pathobiology of asthma. From this perspective, we will provide arguments for and against the positive influence of this molecule in asthma, also indicating the controversies and sketching out the potential directions of research to complete the picture.

## 1. Introduction

Asthma is a clinical diagnosis that covers a variety of phenotypes and endotypes. In the continuous effort towards personalized medicine, remarkable progress has been made to characterize the specific pathobiological marker or mechanism which best defines specific endotypes.

The association between obesity and some difficult to treat forms of asthma raised the possibility of low-grade inflammation, impaired metabolism and dysfunctional adipose tissue secretion as pathogenic mechanisms. The link between disturbed metabolism and inflammation is substantiated by the considerable participation of lipids in the immune reaction in the lung, as components of the immune cell membranes, of proinflammatory eicosanoids and of anti-inflammatory molecules (resolvins and lipoxins) [1].

Adipocytes have a large secretion panel including molecules with dual function in metabolism and in the modulation of inflammation. An excess of adipose tissue has deleterious effects as hyperinsulinemia, which increases airway hyperreactivity, shifts T lymphocytes to the Th2-type response and promotes mast cell degranulation and airway remodeling [1].

Among the numerous molecules secreted by adipocytes, adiponectin (ADPN) is associated with the healthy obese phenotype. ADPN increases sensitivity to insulin, has cardio and vascular protection actions and modulates immune responses. Studies related to ADPN in asthma are far fewer than those dedicated to diabetes or cardiovascular or renal diseases, but this “*versatile player of the innate immunity*” [2] has shown a beneficial role in this pathology. In the current review, we will evaluate this possibility by examining pro and con arguments.

## 2. General Considerations

ADPN is the most abundant adipokine secreted by adipose tissue, described for the first time in 1995, by Scherer et al. [3]. Based on the structural resemblance to complement factor C1q, it was initially named “*adipocyte complement-related protein of 30 kDa*” [3]. Structural similarities with collagens (VIII and X) and tumor necrosis factor alpha (TNFα) were consequently discovered. 

The current name reflects its origin (adipo from the Latin adipous) and its capacity for binding (Latin, *necto*) among its monomers and to a variety of other chemical compounds [4]. The human ADPN molecule consists of 244 amino acids and has four regions: an amino-terminal sequence, a short hypervariable region, a globular domain and a collagen-like domain [5].

Transcription of the ADPN gene is upregulated by several adipogenic transcription factors such as nuclear receptor peroxisome proliferator-activated receptor γ (PPARγ), CCAAT/enhancer-binding protein (C/EBP)-α and -β, forkhead box O1 protein (FOXO1), sirtuin 1 (SIRT1) and SP1 transcription factor (Sp1) [6]. Post-translational modifications take place in the adipocyte cytoplasm to facilitate the tri- and hexamerization of the monomers and, further on, its functionality. Monomers assemble into trimers (which correspond to the low molecular weight (L*M*_W_) forms), hexamers (which correspond to the medium molecular weight (M*M*_W_) forms) and complex, high-order (12–18) multimers (which correspond to the high molecular weight (H*M*_W_) forms) [5].

The excretion of ADPN relies on its binding to internal chaperones, mainly to endoplasmic reticulum p44 (Erp44). Through their binding to Erp44, the ADPN oligomers are retained inside the endoplasmic reticulum. Endoplasmic reticulum oxidoreductin 1 (Ero1) competes the ADPN–Erp44 binding and maintains the secretion. It has been shown that the inhibition of SIRT1 and/or activation of PPARγ leads to increased expression of Ero1-Lα [7]; as these transcription factors are regulated by the nutrition status, it has been suggested that nutrient intake controls the ADPN secretion. 

In the steady state, the ADPN-containing vesicles are located in the trans-Golgi network and traffic through the Golgi/trans-Golgi network to be exported outside the cell [8] after insulin [9], cAMP [10] or β3-adrenergic stimulation [11]. It has been shown that in highly fed mice, who develop obesity, the low level of ADPN is related to a reduction in the expression of β3-adrenergic receptors on adipose cells and the downregulation of the exchange protein directly activated by cAMP [12]. Restriction of calories and weight loss are not able to reverse the secretion [13]. However, it should be mentioned that, in obese patients, the distribution of the ADPN isoforms in plasma (lower H*M*_W_ and higher L*M*_W_ ADPN) is different from isoforms obtained from cultured cells collected by needle biopsy of the SAT from the same patients. As H*M*_W_ forms are generally considered to have a higher biological activity [14], the extrapolation of experimental cell culture studies to humans still needs confirmation.

Several inflammatory proteins inhibit the translation and/or secretion of ADPN: IL-6 [15] and C-reactive protein [16] downregulate mRNA expression and reduce ADPN secretion in a dose-dependent manner. Endothelin 1 acts in differently: it downregulates the expression but increases the secretion of ADPN [17].

## 3. Physiological Role

### 3.1. Main Roles of ADPN

ADPN is considered an insulin sensitizer and a regulator of energy homeostasis. 

In adipose cells, ADPN increases glucose utilization [18] and fatty acid oxidation [19]. In hepatic cells, ADPN suppresses the production of glucose [20]. The same metabolic effects have also been observed in muscle cells [21]. Overall, these results explain the increase in insulin sensitivity.

However, contradictory outcomes have been noticed in skeletal muscle regeneration, contractility and adaptability; these were extensively reviewed elsewhere [22]. 

The L*M*_W_ and M*M*_W_ forms of ADPN cross the blood–brain barrier and have been credited as influencing autonomic functions and feeding behavior. It is still under debate in what direction ADPN influences energy homeostasis; arguments derived from animal studies showed that ADPN both stimulates [23] and inhibits [24] energy intake and/or expenditure. Caloric restriction [25,26] has shown a positive effect on ADPN secretion. Adding the metabolic effects on muscular and adipose tissue [27], the overall conclusion would be that ADPN increases energy efficiency and conservation.

The role of ADPN in inflammation is tissue specific. In the endothelium and adipose tissue, ADPN has anti-inflammatory effects [28,29], while in the synovia and intestine, the end result is a proinflammatory one [30,31].

ADPN polarizes the transformation of peripheral monocytes to anti-inflammatory (M2) macrophages [32], amplifying the response to IL-4 and mediating the decrease in proinflammatory molecules such as TNFα and MCP-1 in M1 macrophages [33,34]. These effects were not reproduced in another study which showed that the effects of ADPN depend on the polarization of the cell; in M1 macrophages, ADPN triggers the expression of proinflammatory cytokines (IL-6, TNFα, IL-12), whereas in M2 macrophages it induces the expression of IL-10 [35,36] and IL-1 receptor antagonist [37]. IL-10 secretion is further enhanced by ADPN’s effects on regulatory T lymphocytes (Tregs) [38].

A possible explanation for these contradictory effects might be the time-dependency response of macrophages to the external stimuli. In the first 24 h after stimulation, ADPN induces the expression of the majority of the M1 macrophage markers (including TNFα and IL-6) and only of a minority of the M2 markers; the expression of the majority of the M2 markers is significantly reduced [39] during this period and is followed, after 3 days, by an increase in Th1 orientation of CD4^+^ T lymphocytes, associated with a response in IFNγ mRNA expression. In contrast, prolonged stimulation led to an increase in macrophage resistance to this stimulus, with a reduction in inflammatory cytokines [40].

### 3.2. Mechanism of Action

ADPN binds to four types of receptors: AdipoR1, AdipoR2, T-cadherin and calreticulin. AdipoR1 has a wide distribution, including in lung cells. AdipoR2 is present mainly in hepatic cells. T-cadherin primarily binds the L*M*_W_ and H*M*_W_ isoforms of ADPN [41] and functions as a reservoir for circulating forms in the endothelium and heart [42]. ADPN also binds to calreticulin to opsonize apoptotic cells on the surface of macrophages and to facilitate efferocytosis [43].

The intra-cellular signal of ADPN is transmitted through AMP-activated protein kinase (AMPK), p38 mitogen-activated protein kinase (p38 MAPK) and PPARα. The last two are activated either directly or as part of the AMPK cascade. Binding to AdipoR1 induces the most significant signal. AdipoR2 has a more reduced effect on the AMPK signaling path but is able to activate PPARα. The absence of T-cadherin inactivates the AMPK signal initiated by AdipoR1/R2, reducing apoptosis [44] and the metabolic effects [45].

AMPK is a key regulator of energy, oxidative status and metabolism [46]. Activated AMPK also phosphorylates proteins involved in autophagy, mitochondrial function and cell growth [47] and blocks several inflammatory pathways related to IL-1β or TNFα activation [48]. The secretion of several cytokines (MCP-1, CXCL10 and CXCL1) was also blocked by ADPN via the AMPK pathway [48].

The metabolic effects of ADPN might have a role in the immune response. The differentiation of CD4^+^ lymphocytes in effector or regulatory subsets is associated with distinctive changes in the metabolic status: while activated T effectors produce their energy mainly by anaerobic glycolysis, Tregs are dependent on AMPK fatty acid oxidation [49].

The induction of eNOS and suppression of iNOS mediated by AMPK were also detected in the lung [50], representing a way to mitigate nitrative stress, as in the arterial vessels [51,52,53].

ADPN activates, directly or through the AMPK, the two isoforms of p38 MAPK in the skeletal muscle [54], in contrast to TNFα, which activates only the p38 α form. This difference seems to be responsible for their contrasting effects. However, p38 MAPK α and β have many other protein interactions in the nucleus or cytoplasm, leading to cell cycle progression, differentiation, chemotactic signals, glycogen synthesis and coactivation of PPARγ [55]. Subsequently to AMPK/p38 MAPK activation, PPARα is translocated to the nucleus of the skeletal muscle cells [56], where it regulates the transcription of genes related to glucose and fatty acid metabolism [57,58].

In PPARα-deficient mice, the inflammation induced by arachidonic acid and leukotriene B4 (LTB4) is prolonged [59]. LTB4 activates PPARα, while PPARα promotes the intracellular breakdown of LTB4, reducing the secretion and balancing the inflammation process towards its resolution [60]. The inhibition of PPARα [61] increases the inflammatory response in macrophages. In contrast, activators of PPARα reduce the cell infiltrate [62] and the secretion of proinflammatory cytokines [63]. Nevertheless, these effects might be mitigated by the negative effect of TNFα, IL-1β and IL-6 on PPARα transcription [64]. Furthermore, PPARα agonists protect against the inhibition of relaxation induced by irritants [65] in the airway smooth muscle (ASM) and upregulate eNOS [66]. 

In obese individuals, PPARα is downregulated in peripheral leukocytes [67], thus limiting the action of ADPN on these cells. However, in the lung of obese and lean rats it has similar expression levels and only PPARγ was found to be markedly elevated [68].

ADPN shares with PPARγ insulin sensitization [69], the rise in eNOS [70], the polarization of macrophages towards the M2 phenotype and the suppression of proinflammatory cytokines [71]. There is evidence that PPARγ regulates ADPN mRNA levels [72] and the expression of ADPN receptors [73]. The binding of PPARγ to the ADPN promoter is inhibited by inflammatory cytokines, such as TNFα [74]. PPARγ directly represses the transcription of ERp44 [75], leading to higher formation and secretion of the H*M*_W_ compound. Clinical data support these findings: a meta-analysis showed that ADPN in patients treated with thiazolidinediones for type 2 diabetes was about 1 standard deviation higher than in controls [76]. This might be the reason why lobeglitazone, which has the benefits of activating both PPARα and PPARγ [77], had such extensive effects on asthma (reduction in inflammatory infiltrate, hyperresponsiveness, mucus secretion).

### 3.3. Circulating ADPN

In healthy subjects, ADPN accounts for 0.01–0.05% of the total plasma protein; regardless of the short half-life, which is approximatively 75 min [78], ADPN is tethered by the T-cadherin receptor in the blood vessels and possibly cleaved to the L*M*_W_ form [79] before it passes into hepatic and renal cells. While the L*M*_W_ form passes through the glomeruli and can be detected in the urine of healthy individuals, renal failure reduces the excretion of ADPN, particularly of the H*M*_W_ form [80]. The balance between secretion, removal and excretion gives a normal value of 3–30 mg/L [81]. The values are lower in healthy, non-obese children and adolescents (median value ranges between 2.5 and 5.2 mg/L) [81] and higher in women, such as in elderly women [82], during pregnancy [83] and in women with a central distribution of fat [84,85]. The circulating level is lower during the luteal phase of the menstrual cycle [86] and in some forms of cancer [87].

High ADPN levels were associated with high N-terminal prohormone of B-type natriuretic peptide (NT-pro BNP) [88], a distinctive marker of cardiac failure related to cardiovascular mortality [89], bone loss [90] and sarcopenia [91].

Among all controversies related to ADPN, there is an overall consensus on the reduction in ADPN secretion in obesity. Low levels of circulating ADPN are also associated with high risk for diabetes [92], cardiovascular disease [93] and chronic kidney disease [94]. A causal relation between ADPN level and the associated diseases was evaluated in a Mendelian randomization study [95]. This study confirmed that ADPN was associated with a healthier metabolite profile (low VLDL, high HDL, low small HDL, low TG, mean particle diameter of VLDL, ApoAI, low TG, low levels of glucose, insulin, lactate and pyruvate, free branched amino acids, saturated fatty acids and systemic inflammatory markers, blood viscosity, higher acetoacetate and high glutamine). However, the Mendelian randomization did not show a direct relation between ADPN and any of these metabolic markers. The only factors which were not rejected by the analysis were inflammatory markers (IL-6 and fibrinogen). The authors considered that the explanation for the observed associations was either a form of reverse causality or the result of a residual confounding (obesity, for example).

## 4. Adiponectin and Asthma

### 4.1. Epidemiological Studies

A well-designed longitudinal study [96] showed that an ADPN level lower than 7 mg/L predicts asthma in women. Compared to previous studies, this one was much larger and was not influenced by gender differences in ADPN secretion, and therefore is frequently considered as a reference.

We reviewed the PubMed database after the publication of this article. Five longitudinal studies and 27 cross-sectional studies were identified (Table 1). Among these studies, low ADPN was found more frequently [97,98,99,100,101,102] than high ADPN [103,104,105] in asthmatics, but most of the studies did not focus on ADPN, which led to a considerable heterogeneity, making comparisons very difficult.

Some reports found no association at all with asthma prevalence, for example, in the elderly population [115]. In asthmatics, there was also a lack of association between ADPN and atopy [106], lung function [106,113] or obesity [114]. 

The longitudinal studies followed morbidly obese patients undergoing bariatric surgery, or the evolution and exacerbations of asthma. The first group of studies has the disadvantage of interfering with the overall effect of weight loss on the evolution of ADPN secretion. Two bariatric patient studies [106,107] showed a favorable effect of weight loss on the recovery of the serum levels of the ADPN after 12 months, which was also found in the general population, after weight loss [110]. In a population of adolescent asthmatics, 1 year of follow-up, after weight reduction, showed an improvement of the ADPN/leptin only in those with moderate or massive weight loss [109]. 

The cross-sectional studies also revealed some controversial results, from low ADPN in obese asthmatics compared to non-obese asthmatics [100] or in asthmatics, regardless of the nutritional status [98,101], to high levels of ADPN associated with asthma prevalence [105]. Other studies found no differences between asthmatics and controls [125], or only lower level of ADPN in obese asthmatics as compared to the non-obese controls [102]. 

In non-obese patients, asthma was associated with higher levels of serum ADPN as compared to healthy controls [104]. According to fat localization, the serum ADPN was inversely associated with BMI and subcutaneous fat [99] and positively associated with epicardial fat.

The relation of ADPN with exacerbations and the severity of asthma raised another series of controversies. Low levels during exacerbations [123] or with severity [118,124,126] were found. In other studies, the broader dysfunction of the adipose tissue was more specifically connected to asthma, as reflected by the relation between ADPN and inflammatory adipokines, mainly leptin [126] or resistin [122]. Additionally, there were also reports of no correlation [120,121].

The differences between these findings might be related to age, gender and race-related variations in ADPN. However, they could also be caused by the ill-defined trajectory of ADPN from serum to bronchial epithelium and lung cells, resident or migrated from the circulation, involved in immunity defense. In this respect, ADPN serum levels might not be the ideal biological product to measure in asthmatics. There are at least two types of arguments for this: one study which recruited young adults, who were non-smokers, found that low serum L*M*_W_ ADPN was better related to asthma than two of the metabolic syndrome components (waist circumference and HDL-cholesterol) [116]. Another study found that the L*M*_W_ form was increased, but the M*M*_W_/total ADPN was the relevant marker [117].

We might assume, by analogy with what has been described for the central nervous system, where only the L*M*_W_ ADPN passes through the blood–brain barrier, that only this form migrates inside the lung interstitium and cells and is able to induce its protective effects. This, for now speculative, mechanism needs to be proven in the future. Another argument might come from research carried out on sputum ADPN in asthmatics. It showed [128] that there is no direct correlation between sputum and serum ADPN, on the one hand, and between sputum ADPN and BMI on the other. Both these findings suggest local specific concentrations of ADPN in the lung. These authors also identified a lower level of total sputum ADPN in asthma patients, compared to controls. Complementary to this, Dorevitch et al. [129] found ADPN to be associated with the total antioxidant capacity of the lung. Taken together, these findings raise the hypothesis that either a deficient level or a deficient uptake and/or utilization of ADPN in the lung are the events most probably related to asthma. In this respect, an occupational asthma study [127] revealed that, after specific challenge with plicatic acid (to confirm asthma related to western cedar wood exposure), ADPN was increased in sputum and this level gradually diminished within 24 h.

An interesting analysis of the local ADPN modifications after bariatric surgery and weight loss was performed by Sideva et al. [108]. Before surgery and weight loss, there was no difference in BAL and serum ADPN, but there was a reduced expression of ADPN receptors in VAT and in epithelial bronchial cells in asthmatics. These changes remained stable 12 months after the intervention only in asthmatics, supporting an independent mechanism for ADPN regulation as compared to the ones strictly related to obesity. 

### 4.2. Mechanistic Studies

From the extensive literature addressing ADPN’s cellular actions, we have selected only the reports which are relevant for the pathophysiology of asthma. 

#### 4.2.1. Dysfunctional Airway Epithelium

Bronchial epithelium integrity protects against air pollution, allergens or pathogens. In asthma, dysregulation and even disruption of the integrity of this natural barrier increases the chance for sensitization and facilitates the action of asthma triggers, thus aggravating disease evolution. ADPN inhibits apoptosis after cell injury and promotes repair and proliferation of the basal bronchial epithelial cells [130]. ADPN also suppresses TNFα expression induced by LPS by autophagy [33] and impedes some potential lesion effects of TNFα on airway epithelial cells. For example, in human primary bronchial epithelial cell cultures, ADPN reduced the secretion of chemokines for monocytes/macrophages (CCL2) and mastocytes (CXCL1), chemokines upregulated by TNFα [131], thus limiting inflammation. In vascular cells, ADPN counteracts the TNFα-related expression of intercellular adhesion molecule-1 and promotion of oxidative/nitrative stress [132,133]. In ADPN double knockout mice, the high resting alveolar macrophage production of TNFα is suppressed by ADPN [133]. In addition, the latter study revealed another benefit of ADPN, i.e., the suppression of matrix metalloproteinase 12 (MMP-12) production. MMP-12 mediates the degradation of the extracellular matrix and is associated with hyperresponsiveness [134] and more severe asthma [135]. 

Whether these experimental effects are transposable in real life is a matter of debate, as the relation between TNFα and ADPN is more complex than in cellular experiments. In M1 macrophages, ADPN induces secretion of proinflammatory cytokines, including TNFα, IL-6 and IL-12 [35]. On the contrary, in adipocytes, the major source of ADPN, TNFα inhibits the transcription of ADPN [136] and suppresses the multimerization of ADPN [137], which might decrease the overall effects of ADPN. An interesting study [138] linked the biological mechanisms of secretion of ADPN with clinical data. The mechanistic part of the study was based on the already known inhibitory effect of TNFα on ADPN secretion [137,139] and revealed that eicosapentaenoic acid (EPA) suppressed the palmitate- and LPS-induced increase in TNFα mRNA expression and NF-κB activation in macrophages. These results were complemented by a case–control trial, in which the supplementation of the diet with eicosapentaenoic acid led to an improvement of the metabolic profile and of ADPN plasma levels [138]. Furthermore, it was shown that the oxidized forms of EPA stimulate PPARγ [140] which, in turn, enhances the transcription of ADPN.

Air pollutants, such as ozone, disrupt the airway barrier and contribute to a high rate of exacerbations in asthma [141]. An argument for a protective effect on ADPN comes from a study on ADPN-deficient mice exposed to subacute ozone levels, in which neutrophil inflammation and the content of protein in BAL (as marker of cell destruction) were significantly increased [142] and positively associated with the infiltrate of interstitial macrophage secretion IL-17. As these modifications were not present in wild-type mice, it was assumed that ADPN was responsible for them.

#### 4.2.2. Hyperresponsiveness of the Airway Smooth Muscle

AdipoR receptors were identified in human airway smooth muscle (ASM). In asthma, ASM is more responsive to external stimuli and has an unbalanced proliferation and apoptosis rate. In severe asthma, ASM even becomes hypertrophic [143] and secretes cytokines and extracellular matrix proteins participating in the remodeling process of the airways. 

An altered response (enhanced and/or prolonged contraction) of the ASM to external stimuli is characteristic for asthma. Its prolonged contraction is due to a decrease in the activity of SERCA2, the carrier of Ca^2+^ into the sarcoplasmic reticulum [144], while globular ADPN is able to upregulate the SERCA in myocytes, at least during ischemia/reperfusion injury [145].

Another mechanism of hyperresponsiveness is the non-adrenergic, non-cholinergic inhibition of the relaxation induced by irritants in the respiratory tract. In this respect, PPARα agonists were protective against such an effect, after exposure to ammonium persulfate [65].

A panel of cytokines modulate responsiveness of the ASM and, among them, IL-13 plays an important role. IL-13 increases the expression of histamine 1 and of cysteinyl leukotriene CysLT1 receptors [146] and mediates the phosphorylation of signal transducer and activator of transcription 6 (STAT6) and mitogen-activated protein kinases (MAPKs), augmenting the Ca^2+^ response to histamine [147]. As a consequence, IL-13 enhances the potency of histamine, carbachol and leukotriene D_4_, as contractile agonists, in ASM. ADPN overexpression is able to counteract IL-13 actions in mice, after ovalbumin acute exposure [148]. In this experiment, overexpression of ADPN was also able to decrease the expression of omentin and arachidonate 15-lipoxygenase. It is notable that in allergic asthma, omentin [149,150] and arachidonate 15-lipoxygenase are both upregulated [151]. Therefore, ADPN’s capacity to neutralize the hyperresponsiveness of the ASM could be mediated indirectly by the downregulation of omentin and arachidonate 15-lipoxygenase, having as a consequence a reduction in mucus production, inhibition of CXCL-10, 15-Hydroxyeicosatetraenoic acid (15-HETE) and hydroperoxy-eicosatetraenoic acid (15-HETE-PE) or eotaxin production [152,153], other contributors to allergic inflammation.

In asthma, ASM proliferates and the thickness of the ASM layer increases. This proliferation is a characteristic of the disease [154] which contributes to the aggravation of the evolution even in the absence of massive concurrent inflammation. 

In ASM, the AMPK signal suppresses the smooth muscle proliferation via the mTOR pathway [155] and inhibits the proliferative effect of transforming growth factor β1 (TGF-β1) [156]. ADPN is known to stimulate AMPK in vascular smooth muscle [157] and to suppress pulmonary artery remodeling [158]. Even if we cannot extrapolate the findings regarding the vascular smooth muscle to the ASM, ADPN remains a candidate for further investigation of this effect.

Prevention of the proliferation of ASM could be another consequence of the inhibition of TNFα, because TNFα induces the proliferation of ASM through SOX-18-Notch1 signaling [159]. Notch1 promotes Hes1 expression which, in turn, downregulates the phosphatase and TENsin homolog (PTEN) [160]. PTEN is a regulator of cycle cell progression and blocks proliferation through inhibition of the phosphoinositide 3 kinase/Akt pathway. Indeed, silencing of PTEN reduced ASM proliferation in asthma [161]. As ADPN interferes with TNFα secretion, it would be useful to investigate the pertinence of this possible ADPN effect on the expression of PTEN and, ultimately, on the inhibition of human ASM proliferation.

In addition to the anti-inflammatory role, PPARα agonists also protect against the non-adrenergic, non-cholinergic inhibition of relaxation of ASM induced by irritants [65].

#### 4.2.3. Enhanced Mucus Hypersecretion

In the previously mentioned experiment [148], ADPN overexpression interfered with mucus secretion by inhibiting the expression of omentin and MUC5AC. Omentin is mainly expressed in goblet cells and correlates to Th2 markers [151]. MUC5AC is secreted by goblet cells in the surface epithelium and in the terminal secretory ducts of submucosal glands, in proximal and distal airways [150]. In MAC5AC mucin-deficient mice, the mucus occlusion was considerably reduced and the hyperresponsiveness to ovalbumin abolished [162]. Downregulation of these two genes by ADPN overexpression, if also proven in human airways, would indicate another benefit of ADPN in asthma. 

#### 4.2.4. Inflammation and Immune Response

The mechanism of allergic asthma starts with the uptake of allergens by epithelial cells, followed by the secretion of cytokines to activate dendritic cells and type 2 innate lymphoid cells (ILC2s). Dendritic cells migrate to lymph nodes and present allergens to naïve lymphocytes, promoting their differentiation to Th2 cells. Th2 cells and ILC2s will further secrete the Th2 panel of biomarkers (IL-4, IL-5 and IL-13) responsible for eosinophilic inflammation. In this form of asthma, activation of Th1 functions acts as a regulator of Th2 activation [163].

The steps of neutrophilic asthma (non-Th2 or Th1 asthma) are not so well defined. A plethora of triggers (endotoxin, ozone, particulates, virus infection, etc.) [164] cause injury to epithelial cells, inducing the release of IL-6, IL-8 and LTB4 to attract neutrophiles and shift immune cells toward Th1 and Th17 helper responses, further increasing IL-8, IL-17, IL-22, IFNγ and TNFα [165]. The regulatory function is attributed mainly to Treg cells, both thymus derived and locally induced. 

Alveolar and interstitial macrophages participate in the inflammation, acquiring different roles during the evolution of the allergic process, for example, during the sensitization step, M1 (IFNγ-induced) macrophages prevent an allergic reaction, while in a later phase they promote eosinophilic inflammation and hyperresponsiveness [166]. Defective phagocytosis and increased inflammasome formation in relation with airborne particles and altered efferocytosis were cited as dysfunctional changes of macrophages in asthma [167]. The concept of differentiation into M1 or M2 types of macrophages has been challenged and, currently, there is more consensus about a polarization spectrum ranging from IL-4-stimulated macrophages (classically known as “alternative”) to IFNγ-stimulated ones (classically known as “activated”), with many intermediate forms [168].

The connection of ADPN with several immunological processes in asthma is summarized in Table 2 and will be briefly described below.

AdioR1 and AdipoR2 are expressed by dendritic cells [170], suggesting a functional role of ADPN in these antigen-presenting cells, which are best suited to initiate the allergic response in the lung. Data regarding the ADPN effects on dendritic cells have been rather contradictory to date. One study showed that bone marrow dendritic cells in contact with ADPN lowered the expression of costimulatory molecules (CD80 and CD86) and markedly or moderately reduced the secretion of IL-12, IL-6 and IL-10, respectively [171]. Moreover, dendritic cells dependent on ADPN increased the expression of programmed death ligand 1 (PDL-1) in T lymphocytes and drove the differentiation of CD4+ positive cells towards Treg. The activation of the PD-1 pathway limits the proliferation of the pulmonary ILC2 through metabolic regulation and production of Th2 mediators [178]. Furthermore, PD-L1 expression after OVA challenge of lung dendritic cells, macrophages and B cells is sufficient to inhibit IFNγ production and to mitigate airway hyperresponsiveness, mucus secretion and inflammation [179]. The importance of PDL-1 is also suggested by clinical studies, in which soluble PDL-1 and allergic rhinitis were negatively correlated [180]. Overall, these effects would oppose sensitization to allergens. In fact, an increase in serum ADPN in mice sensitized to ovalbumin reduced eosinophil infiltrate and Th2 markers, namely IL-13 and IL-5, in BAL [178] and blunted the airway hyperreactivity reaction.

A proinflammatory effect of ADPN on dendritic cells was also mentioned in one study, in which ADPN-activated dendritic cells produced IL-12 and IL-6 and polarized to CD4+ T cell Th1 and Th17 lymphocytes [170]. IL-12 is an inducer of the Th1 response and has been recovered from BAL of asthma sensitized mice chronically exposed to particulate matter [181] and from children with severe neutrophilic asthma [182]. 

Therefore, the effect of ADPN on dendritic cells should be regarded with caution. Possible differences are related to the type of dendritic cells and to the underlying immune response as well; the dendritic activation of the Th2 mechanism may be restricted by ADPN, whereas the Th1/Th17 one may be facilitated.

Sensitization is associated with a lower expression of ADPN receptors (AdipoR1, AdipoR2 and T-cadherin) in the lung [176]. It should be acknowledged that sensitization needs the presence of T-cadherin, as T-cadherin-deficient mice do not develop an allergic response to ovalbumin [42]. In the absence of ADPN, in bideficient mice (in T-cadherin and ADPN), the absence of T-cadherin as a protection factor for sensitization was abolished. The authors concluded that T-cadherin, probably independent of ADPN binding, facilitates migration of inflammatory cells (neutrophils, eosinophils) in the airways, and downregulates the proteic components of surfactants to promote the allergic reaction. However, it must be established if ADPN binding to T-cadherin is able to diminish T-cadherin’s potential for sensitization or not.

Group 2 innate lymphoid cells (ILC2s) are a cell population present in large numbers in asthmatic airways [183] and are one of the drivers of acute type 2 inflammation in the lung [184]. The traffic of ILC2s from marrow to their tissular location is enhanced by IL-33 [185]. AMPK activation induced by ADPN inhibits the IL-33-stimulated NF-κB pathway and IL-13 production in ILC2s [172]. This could also affect the initiation of the Th2 immune response in the lung, as ILC2s are able to act as antigen-presenting cells and to express IL-5 even in a steady state [186]. 

Earlier studies highlighted the mutual suppression between the Th1 and Th2 responses, orchestrated by IFNγ and IL-4, in the differentiation and effector phases [187]. More recent research has shown that in both eosinophilic and neutrophilic asthma, T1 and T2 inflammation coexists in different proportions [188]. Furthermore, the commitment of CD4 + T cells from IFNγ double knockdown mice to Th2 (IL-4, IL-10 and IL-13 secretion) depends on the systemic administration of IFNγ [189]. 

ADPN plays a role in modulating IFNγ secretion in macrophages and lymphocytes. Most studies suggest a negative impact on IFNγ production, but few are dedicated to the alveolar or interstitial lung macrophages. In peripheral monocytes, ADPN promotes the alternative form [32], although, in others, the proinflammatory type was promoted [39]. However, even M2 macrophages are not a homogenous population and this kind of polarization does not necessarily activate the resolution of the inflammation. In some experiments, M2 polarization contributed to the progression of Th2 inflammation via decreasing interferon regulatory factor 4 [190]. 

A demonstration of the lowering effect of IFNγ in the lung, induced by ADPN, comes from a study in which the number of IFNγ-producing influenza-specific T cells was diminished [175]. This finding could be relevant for viral related forms of asthma. There was also an experiment showing that ADPN reduces IFNγ and Th17 cell differentiation and restrains glycolysis in Th1 and Th17 cells [174]. Overall, the findings described above would be beneficial mostly in neutrophilic asthma. Another presumptive action of ADPN in neutrophilic asthma would be the PPARα blockade of LTB4 [60]. In PPARα-deficient mice, the inflammation induced by arachidonic acid and leukotriene B4 (LTB4) is prolonged [59]. The mechanism of this prolongation is a negative feedback loop between LTB4 and PPARα. While LTB4 activates PPARα, PPARα promotes the intracellular breakdown of LTB4, reducing secretion and balancing the inflammation process towards its resolution [60].

In fact, the modulation of PPARα expression by inhibitor or activator molecules leads to significant consequences for asthma. For example, the inhibition by MK-866 (a more selective inhibitor of PPARα as compared with other PPARs) [61] increased the inflammatory response to mono(2-ethylhexyl) phthalate in alveolar macrophages. Fenofibrate, a PPARα activator, reduced the airway cell infiltrate and the inflammatory markers (IL-4, IL-5, TNFα, macrophage-inflammatory protein-2 and monocyte chemoattractant protein-1) in a dose-dependent manner, after ovalbumin challenge in sensitized mice [62]. In another experiment, besides a reduction in IL-4 and TNFα, fenofibrate inhibited IL-17 and IL-23 expression in the lung [63]. The effect was comparable to the one induced by dexamethasone. Nevertheless, these effects might be mitigated by the negative effect of TNFα, IL-1β and IL-6 on PPARα transcription [64], as mentioned above. 

As an effect on macrophage polarization, differentiation of CD4+ cells is modulated in more than one way. For example, instead of reducing IFNγ on polyclonally activated CD4+ cells, as shown above, ADPN increased IFNγ production via the p38 MAPK signal [39]. 

Regarding eosinophilic asthma, several experiments showed ADPN-related benefits. In experimental asthma, the Th2 response was reduced after infusion of ADPN [166]. ADPN was also able to reduce eotaxin secretion [148] and eosinophil chemotaxis and adhesion capacity promoted by eotaxin, via AdipoR [191]. There is also some evidence regarding interference with the chitin-mediated mechanism [185] of eosinophil recruitment. In this latest study, chitin inhalation reduced AdipoR1 receptors in lung leukocytes, particularly in eosinophils; coaspiration of chitin and ADPN significantly decreased the lung eosinophil infiltrate. Furthermore, the process was not restricted to the recruitment of the circulating cells, as pre-incubation of bone marrow-derived eosinophils with ADPN decreased the migration to peripheral tissues mediated by eotaxin. Concordant results were found in APN-deficient mice who presented higher levels of circulating eotaxin and eosinophil chemotactic protein 2 and developed more severe lung eosinophil infiltrates [192].

Many of the positive actions of ADPN concern the anti-inflammatory effect and Treg activation. ADPN restores the emphysema-like phenotype, characterized by increased production of TNFα and matrix metalloproteinase 12 in ADPN-deficient mice [133]. Binding of ADPN to calreticulin brings back the efferocytosis capacity of macrophages [43], a defense mechanism impaired in asthma, which contributes to the chronicization of inflammation and airway remodeling [167]. 

Tregs suppress inflammation by upregulating immunosuppressive molecules (IL-10, TGF-β, IL-35) and cytolytic molecules, depriving lymphocytes of trophic cytokines (IL-2), downregulating tissue receptors and preventing the acquisition of proinflammatory functions in all types of lymphocytes (B and T cells, NK, monocytes), ILC2 cells, antigen-presenting cells, mast cells, eosinophils and neutrophils [193]. In asthma, Treg depletion facilitates sensitization [194] and maintains an active inflammation process [195] and active Th2 response [196]. 

Peripheral Tregs express more AdipoR1 than thymus-derived ones. Globular ADPN increases the secretion of IL-10 in peripheral Tregs, particularly in a Th2 milieu [38], in macrophages, monocytes and dendritic cells [37]. In macrophages, IL-10 induces the mRNA expression of the tissue inhibitor of metalloproteinase 1 (TIMP-1) [36]. The TIMP-1 level is significantly higher in asthmatics compared to controls [197], in association with one or several matrix metalloproteinases. In fact, the TMP-1 increase should be considered as a mechanism to compensate the accelerated turnover of the extracellular matrix produced by the matrix metalloproteinases. In line with this assumption, in TMP-1-deficient mice, the allergic Th2 inflammation is exacerbated [198].

Human Tregs have a different metabolism than conventional Tregs, showing an increased rate of glycolysis and fatty acid oxidation and higher oxygen consumption [199]. Knowing the capacity of ADPN to mobilize cellular energy reserves, mainly to increase fatty acid oxidation and glycolysis via AMPK and PPARα signaling, it would be interesting to explore these mechanisms in Tregs.

In summary, from the mechanistic point of view, both Th2 and Th1 phenotypes of asthma might benefit from ADPN. Interesting regulatory mechanisms have been proposed with positive effects in some experimental studies. Different results were published depending on the types of cells, strain of mice, cell culture environment and interaction with other cytokines. There are indeed few clinical data, therefore the numerous ADPN effects that must be considered a challenge to be carefully handled.

#### 4.2.5. Airway Remodeling

Remodeling of the airways might be considered the final result of the pathological mechanisms in asthma. The complexity of airway remodeling has been recently revised [200] and below we will describe only the processes for which data regarding ADPN influence were found.

The inflammatory process itself and/or the asthma attack triggers (e.g., particles, irritants) create reactive oxygen species, which generate tissue damage, release of endogenous DAMPs and myofibroblast differentiation. All these effects have an impact on epithelial cell dysfunction, ASM proliferation and fibrosis, which are key elements of airway remodeling. Oxidative stress in the adipose tissue reduces the secretion of ADPN [201]. In the lung, ADPN protects against oxidative stress induced by cigarette smoke extract [202] and by airway sensitization in obese mice [193]. NF-κB and inducible nitric oxide synthase (iNOS) are also suppressed by ADPN. The induction of eNOS and the suppression of iNOS in the lung [50] represent ways to mitigate nitrative stress. The AMPK signal upregulates eNOS activity and reduces reactive oxygen species (ROS) [51]. In accordance with this, ADPN suppressed the ROS formation induced by the lipopolysaccharide palmitic acid, in monocyte cellular lines, by AMPK activation [52]. In high-fat diet-fed mice sensitized to ovalbumin, ADPN reduced the markers of inflammation and of oxidative stress [203]. 

Remodeling includes the interstitium and the activity of the matrix metalloproteinases. The level of extracellular matrix metalloproteinase 9 (MM9) in serum was significantly decreased after bariatric surgery, and this decrease was corelated with the increase in ADPN [27]. This might have implications for asthma, as MM9 was also elevated in serum, sputum and BAL, in asthmatics [203]. However, no matter how important MM9 is for asthma, the study mentioned above [27] was not meant to identify whether there was a connection between MM9 and ADPN variation or if it was merely a coincidence, therefore a possible role of ADPN in MM9 remains uncertain.

The AMPK signal suppresses smooth muscle proliferation via the mTOR pathway [154]. In asthma, ASM produces various cytokines, such as monocyte chemoattractant protein 1 (CCL2), which, in turn, promotes fibrocyte migration and ASM hyperplasia in asthma [204]. Downregulation of CCL2 by ADPN potentially prevents this complication of asthma in human primary bronchial epithelial extract [131]. The same protective effects on ASM hyperplasia were obtained from the comparison between wild and ADPN −/− mice with asthma [205]. In this study, intranasal instillation of ADPN in ADPN −/− mice prevented the remodeling of the airways, namely the increase in airway smooth muscle thickness and peribronchial inflammation. 

ADPN inhibits fibrotic gene expression (e.g., for collagen) [206] in cultured fibroblasts. This effect is mediated by the inhibition of transforming growth factor-β (TGF-β) by the AMPK signal [156]. The inhibitory effect is best documented in hepatic tissue [207]. In acute lung injury, PPARα agonists suppressed TGF-β1, accelerating the recovery [208], but in patients with chronic interstitial lung disease, high ADPN and ADPN/leptin levels were associated with exacerbations [209]. 

To date, some positive results in asthma have been communicated, namely on the inhibitory effect of fenofibrate on the phenotypic transition of fibroblasts to myofibroblasts [210]. Another compound with a preventive effect on airway remodeling, BSYQF, acts through complete restoration of ADPN gene expression in the lung [211], but it is not yet clarified which of its multiple effects (inhibition of ASMC proliferation and of peribronchial collagen deposition and restoration of mitochondria functionality) is determined by the expression of ADPN.

#### 4.2.6. Vascular Modifications

An abnormal function of the endothelium contributes to inflammation and tissue damage in asthma. Reduced flow mediated dilatation, increased vascularity, a blunted response of airway blood flow to albuterol, an increased number of progenitor vascular cells after allergen challenge and high levels of angiopoietin and VEGF [212] were reported in asthma. There is no consensus about ADPN’s influence on blood vessels, as both angiogenic and anti-angiogenic effects have been described. In mice with overexpression of ADPN, VEGF in brain microvessels was increased via the AMPK signal [213]. In human coronary artery endothelial cells, ADPN suppressed VEGF’s effects through cAMP/PKA signaling, namely the induction of reactive oxygen species formation and transvascular cell migration [214]. In mice exposed to house dust mites, the inhibition of VEGF signaling lessened the development of inflammation and airway remodeling [215]. However, it remains to be proved whether ADPN acts in the same way in coronary arteries and in lung vessels.

To the best of our knowledge, there are no data related to ADPN’s effect on endothelium dysfunction in asthma. Therefore, it is uncertain how the experimental data presented above translate into benefits for asthma or, on the contrary, accentuate the pathological mechanisms.

#### 4.2.7. Reduction in Respiratory Muscle Strength

Extensive literature has been dedicated to the effects of ADPN on skeletal muscle. ADPN improves the autophagy and regenerative capacity of the muscle, reduces endoplasmic reticulum stress and increases contractility [22]. Nevertheless, different studies have pointed out contrasting results about ADPN’s effects on muscle regeneration [216] on the one hand, and on muscle waste [217] on the other. Ineffective muscular contraction, inadequate muscle strength [218] and a reduction in respiratory muscle mass are important aggravating factors for asthma. The role of ADPN in preventing deterioration or restoring functionality remains to be clarified by future research.

#### 4.2.8. Obesity-Related Asthma

**Figure 1:**
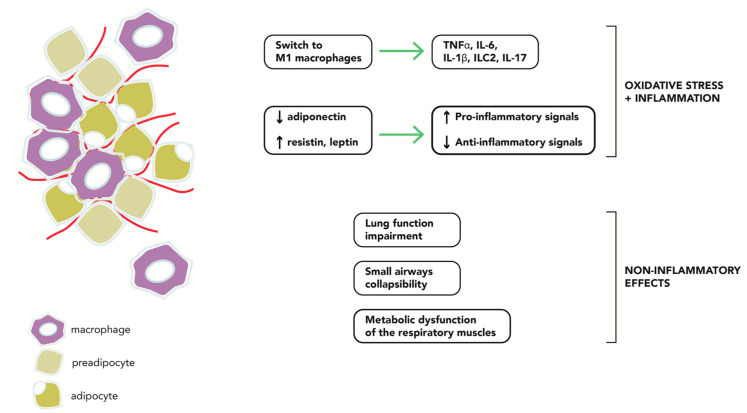
Pathophysiological factors related to the asthma-obese phenotype.

The severity of the asthma–obese phenotype is a consequence of multiple factors (Figure 1). As the majority of ADPN originates in adipose tissue, many studies have investigated this molecule in an attempt to decipher the mechanisms of the asthma–obese phenotype. Indeed, the impaired secretion of soluble factors by obese adipocytes had proinflammatory effects on airway epithelial cells [219], and weight loss restored this balance and attenuated the inflammation. The non-eosinophilic pattern of inflammation and the activation of non-Th2 adaptative mechanisms has shown increasing importance, although eosinophilic inflammation is sometimes present in this form of asthma. 

Most epidemiological studies found low serum ADPN levels in obese asthmatics, particularly in women [124,220]. However, the relation with the severity of asthma, assessed by the degree of bronchial obstruction, was inconstant; both negative [124] and positive [221] relations between ADPN and lung function were found. Reversibility to normal levels of ADPN, after body weight reduction, were correlated with improvement of asthma [100], but did not necessarily imply a resolution of the airway inflammation [222]. The impact of weight loss created a new classification of the obese–asthma phenotype into early- and late-onset obese asthma [223], attributing more influence to inflammation in the first group and to the impairment of the respiratory mechanics in the second one. The different endotypes of the asthma–obese phenotype might explain the lack of interaction between obesity and asthma, in terms of airway inflammation, described in some studies [224], and the distinctive results related to weight loss.

Polymorphisms of the ADIPOQ gene, in particular the G allele of rs822396 and T allele of rs1063537, were associated with an increased risk for asthma, while variants rs11760956, rs11763517 and rs2167270 were protective [225]. Both polymorphisms were related to low ADPN [226] and asthma [123]. Interestingly, the protection was lost in overweight individuals [225]. We do not know if this corresponds to the reduced expression of AdipoR1 in overweight patients and the “recuperation” of normal expression in severe obese patients [178]. In this experimental model [178], AdipoR1 expression in regulatory lymphocytes (Tregs) in the lung was reduced in obese mice but, in excessive obesity, the expression was increased. In allergic obese mice, AdipoR1 was also increased. Notably, both Tregs of the thymus and of peripheral origin were reduced. In OVA-sensitized mice, the AdipoR1^+^Treg level was significantly reduced independent of the body weight.

The inflammatory status generated by adipose tissue is well documented and metabolic inflammation was linked with metabolic syndrome-related diseases, but also with COPD and asthma. A large number of M1 macrophages, CD4 + Th1 cells, CD8 +T effector cells and mast cells and a small number of M2, CD4 + Th2, eosinophils and Tregs were found in adipose tissue from obese individuals, but the systemic effects of these findings are not always straightforward. For example, the high level of tryptase secreted from the mast cells resident in adipose tissue was not correlated with respiratory symptoms [227]. In terms of the type of airway infiltrate, the findings are also very contradictory, ranging from typically eosinophilic to predominantly neutrophilic non-Th2 forms [228].

Animal studies have tried to decipher the substrate of the hyperresponsiveness related to obesity and to the proportion of lean/fat mass. In a study on particulate matter-induced AHR in mice [229], serum ADPN was reduced only in those who gained weight after a high-fat diet and who also showed a higher fat mass for the same weight gain.

In obese sensitized mice and in non-obese asthmatic mice, systemic administration of ADPN was able to reduce lung cell infiltrate and the concentration of TNFα, eotaxin and myeloperoxidase (MPO) in BAL, after ovalbumin challenge [130]. Interestingly, there was no difference between asthmatic obese and asthmatic lean mice in terms of BAL eotaxin concentration, but MPO was higher in the obese mice. Some of these findings contrast with those from a study which compared obese women with and without asthma [230]. In this study, asthmatics had higher serum eotaxin, positively correlated with ADPN. The same positive correlation of ADPN was also found with regulated upon activation normal T cells, expressed and secreted (RANTES), while with IFNγ, the relation was the inverse.

In obese asthmatic mice, a large number of macrophages were found in the lungs [231]. After house dust mite challenge, the expression of total, M1 and M2 macrophage markers and of both Th2 and non-Th2 cytokines increased in the lungs. Considering ADPN’s effects on macrophage polarization [43,167,170], the ADPN-specific influence in house dust sensitization deserves further examination.

Obesity reduces the number of Tregs in lung and adipose tissue [178]. The previously mentioned effects of ADPN on Tregs and on IL-10 secretion could help restore homeostatic balance.

Finally, some arguments on the significance of ADPN in obese asthmatic individuals derive from ADPN agonist utilization. It was demonstrated that ADPN agonists reduce IL-4, TNFα, IL-17 and IL-23 expression in the lung [63]. The magnitude of this effect was comparable to the action of dexamethasone. Nevertheless, these effects might be mitigated by the negative effect of TNFα, IL-1β and IL-6 on PPARα transcription [64]. Another factor of mitigation might be the downregulation of PPARα in obese individuals, a characteristic that was confirmed in peripheral leukocytes [67], but needs to be confirmed in lung cells. An indirect confirmation consists in the detection of a similar expression of PPARα and AdipoR in the lungs of obese and lean rats [68]. Regarding the significance of ADPN modulation of PPARs in asthma, it is notable that PPARα and PPARγ expressions are upregulated in the lung tissue of OVA-challenged obese mice [232].

## 5. Conclusions

ADPN has a great potential to interfere with the pathological mechanisms of asthma. Most of its beneficial effects were demonstrated in animal models and in cell cultures, with inconsistencies related to the type of asthmatic inflammation, cells and methodology of the experiment. 

Clinical data rely mainly on the association of serum ADPN with various unfavorable types of evolution in asthma in a few prospective cohorts. As regards the obesity phenotype, it also seems that ADPN (and probably other adipokines) has beneficial effects in a subgroup of asthmatics.

There is a certain need to clarify the implication of ADPN in the complex and heterogenous inflammatory process of asthma and to adequately investigate the diverse endotypes of asthma. Although some promising results have been published to date, ADPN has many unknowns to be revealed in order to accurately identify its significance for asthma.

## Figures and Tables

**Table 1:** Association of adiponectin and asthma in epidemiological studies.

Study	Type	Study Population	Directionality of Asthma—ADPN Relation	Main Results
Baltieri, L. et al., 2018 [106]	Longitudinal	80 bariatric surgery patients with asthma	↑ ADPN parallel to clinical status Association with clinical status and indirect relation with weight loss	↑ ADPN in parallel with weight loss and clinical status improvement No lung function improvement after 1 year of follow-up.
van Huisstede, A. et al., 2015 [107]	Longitudinal	27 bariatric surgery patients with asthma (BS + A) compared with 12 obese with asthma without bariatric intervention (NBS + A)	In relation with weight loss	↑ ADPN in BS + A and did not change in NBS + A after 1 year of follow-up
Sideleva, O. et al., 2012 [108]	Longitudinal	11 obese women with asthma and 15 obese women without asthma bariatric intervention	No association with weight loss	At baseline: APDN in visceral fat significantly lower in asthma After 12 months: (a) no modification in ADPN expression in SAT (b) no modification of AdipoR1, AdipoR2 and T-cadherin expression in bronchial epithelial cells (c) no significant difference in BAL ADPN (d) BAL ADPN not correlated with VAT ADPN
Leão da Silva, P. et al., 2012 [109]	Longitudinal	84 asthmatic adolescents, follow-up 1 year after a weight loss intervention	↑ ADPN parallel to clinical status Association with clinical status	↑ ADPN and ADPN/leptin (A/L) ratio and improved asthma symptoms after moderate (8–14 kg) and massive (>14 kg) weight loss
Abd El-Kader, M.S., 2013 [110]	Longitudinal	80 obese asthmatic children	Indirect relation with weight loss	↑ ADPN in serum after weight loss in obese asthma (8 weeks of follow-up)
Tsaroucha, A. 2013 [111]	Longitudinal	32 stable adult asthmatics, 37 asthmatics during exacerbation, 22 controls	Indirect relation with severity and exacerbations	↓ ADPN in severe asthma ↓ ADPN during exacerbations
Mikalsen, I.B. et al., 2020 [112]	Cross-sectional	384 adolescents with atopy	No association with sensitization or lung function	No association between ADPN and lung function and atopic sensitization
Huang, F. et al., 2017 [113]	Cross-sectional	28 obese adolescents with asthma, 46 obese adolescents without asthma, 58 normal-weight adolescents with asthma and 63 healthy controls	Lower levels in obesity but no relation with lung function in asthmatics	↓ H*M*_W_ ADPN in obese subjects than the normal-weight subjects with or without asthma H*M*_W_ ADPN not correlated with ↓ FEV_1_/FVC ratio
Grotta, M.B. et al., 2013 [114]	Cross-sectional	16 asthmatic obese, 16 asthmatic non-obese, 5 non-asthmatic obese, 5 non-asthmatic non-obese	No differences in asthma and in obesity	Levels of ADPN did not significantly differ among these groups
Song, W.J. et al., 2012 [115]	Cross-sectional	994 participants from the general population, age ≥65 y	No association with asthma	No association between asthma and serum ADPN levels
Barton, J.H. et al, 2016 [97]	Cross-sectional	HIV patients, 33 with asthma and 23 with COPD	Lower level in asthma compared to COPD	↓ ADPN in asthma vs. COPD
Lu, Y. et al., 2016 [98]	Case–control	79 asthma versus 69 controls	Lower ADPN in asthma	↓ ADPN in asthma No direct interaction with BMI was checked, but the group with asthma had higher BMI
Capelo, A.V. et al., 2006 [99]	Cross-sectional	83 asthma patients, women	Indirect relation with BMI in asthmatics	ADPN serum level inversely associated with BMI and SAT
Magrone, T. et al., 2014 [100]	Cross-sectional	80 children: 17 non-asthmatics and non-obese, 19 obese, 28 obese asthmatics and 16 non-obese asthmatics	Lower ADPN in obese asthmatics	↓ ADPN in serum in asthmatic obese compared to non-obese asthmatics and controls
Aydin, M. et al., 2013 [101]	Case–control	45 asthmatic vs. 30 healthy controls	Lower ADPN in asthma	↓ ADPN levels lower in asthma patients compared to controls
Jensen, M.E. et al., 2013 [102]	Case–control	361 particpants: 74 obese asthma, 249 non-obese asthma, 9 obese control, 29 non-obese control children	Lower ADPN in obese asthmatics	↓ ADPN in obese asthma versus non-obese controls No difference in ADPN between obese and non-obese asthma
Carpio, C. et al., 2016 [103]	Case–control	25 asthma, 23 dyspnea perceived by patients as asthma (misdiagnosed), 27 controls	No significant relation	Higher (but not significantly) ADPN in asthma compared to control and misdiagnosed (self-reported) asthma
Ozde, C. et al., 2015 [104]	Case–control	68 non-obese asthma children and 39 controls	Higher ADPN in asthmatics	↑ ADPN in asthma compared to controls, associated with higher epicardial adipose tissue
Lu, Y. et al., 2015 [105]	Case–control	70 asthmatics and 69 controls	Higher ADPN in asthmatics	↑ ADPN in asthma (both obese and non-obese groups comparison) A positive association with asthma prevalence was observed for medium and high tertiles of ADPN
Matsumoto, Y. et al., 2013 [116]	Cross-sectional	79 young adults (14 asthmatics/62 non-asthmatics), non-smokers	Lower H*M*_W_ and L*M*_W_ ADPN in asthmatics	↓ M*M*_W_ and L*M*_W_ ADPN in asthmatics Differences in H*M*_W_ ADPN were not significant Multivariate regression analysis of the M*M*_W_ and L*M*_W_ ADPN levels, asthma was found to be more significantly associated with the L*M*_W_ ADPN level than age, waist circumference or HDL-C
Hayashikawa, Y. et al., 2015 [117]	Case–control	61 asthmatics versus 175 controls	Higher L*M*_W_ ADPN and lower M*M*_W_ in asthmatics	Significantly ↑ L*M*_W_ ADPN level and significantly ↓ M*M*_W_ /total ADPN in asthmatics vs. controls after adjustments for confounding factors in both sexes
Ma, C. et al., 2019 [118]	Cross-sectional	122 children with asthma	Lower ADPN in severe asthma	↓ ADPN in severe asthma Asthma severity was positively correlated with BMI
Ceylan, E. et al., 2019 [119]	Case–control	120 asthmatic patients (47 with comorbidities and 73 without comorbidities) + 35 healthy controls	No association	No difference in ADPN in asthmatic and controls No difference in ADPN in asthmatics with or without comorbidities
Kalmarzi, R. et al., 2017 [120]	Cross-sectional	90 asthmatic women	Leptin/ADPN levels correlated positively with asthma severity	(a) No correlation between ADPN level with asthma severity and BMI (b) Serum leptin and leptin/ADPN levels correlated positively with asthma severity and BMI and correlated inversly with FEV1 and FVC
Zhang, X. et al., 2018 [121]	Cross-sectional	108 asthmatic patients	No relation with obesity and asthma control	No relation between ADPN, obesity and asthma control
Ballantyne, D. et al., 2016 [122]	Case–control	96 asthmatics and 46 controls	High resistin:ADPN ratio associated with severity	↑ Resistin:ADPN ratio in asthma and in severe vs. mild-to-moderate asthma
Ding, Y. et al., 2015 [123]	Case–control	120 asthmatics and 120 controls, adults	Low ADPN during exacerbations	↓ ADPN in acute exacerbation A positive association between the rs1501299 SNP of the ADIPOQ gene and acute exacerbation
de Lima Azambuja, R. et al., 2015 [124]	Cross-sectional	75 asthmatic patients (25 in each BMI category)	Lower ADPN with increased BMI in asthmatics Indirect association of ADPN and lung function	↓ ADPN serum level with increase in BMI Higher ADPN levels associated with lower FEV_1_ and FVC
Dogru, M. et al., 2015 [125]	Cross sectional	82 non-obese asthmatic children and 28 controls	Lower ADPN in uncontrolled asthma	ADPN levels in non-obese asthmatics were not different from controls ↓ ADPN associated with uncontrolled asthma
Newson, R.B. et al., 2014 [126]	Cross-sectional	1370 patients: 532 with asthma only, 522 with chronic rhinosinusitis only, 316 with asthma and chronic sinusitis and 585 controls	Leptin/ADPN ratio positively associated with the severity of asthma	Leptin/ADPN ratio positively associated with the severity of asthma, especially in females
Biagioni, B.J. et al., 2014 [127]	Cross-sectional	19 asthmatic patients	Increase in sputum ADPN after specific inhalation challenge with plicatic acid	↑ sputum ADPN after plicatic acid challenge, particularly in non-obese
Sood, A. et al., 2014 [128]	Case–control	44 asthmatics and 44 controls	Inverse relation of sputum ADPN with asthma	(a) Sputum total ADPN was not correlated with serum ADPN or BMI (b) ↓ Sputum total ADPN among asthmatics than controls (c) ↑ Sputum total ADPN associated with ↓ odds for asthma, even after adjustment for systemic adiposity measures including serum ADPN
Dorevitch, S. et al., 2013 [129]	Cross-sectional	350 non-smoking adults and children	Direct relation with the total antioxidant capacity in exhaled breath	ADPN associated with total antioxidant capacity in exhaled breath

ADPN = adiponectin; BS + A = bariatric surgery patients with asthma; NBS + A = non-bariatric surgery patients with asthma; BAL = broncho-alveolar lavage; VAT = visceral adipose tissue; SAT = subcutaneous adipose tissue; L*M*_W_ ADPN = low molecular weight adiponectin; H*M*_W_ adiponectin = high molecular weight adiponectin; M*M*_W_ ADPN = medium molecular weight adiponectin; HDL-C = high-density lipoprotein cholesterol; FEV1 = forced expiratory volume in the first second; FVC = forced vital capacity; BMI = body mass index. ↑= increase level; ↓= decrease level.

**Table 2:** Cellular mechanisms of adiponectin relevant for asthma.

Cell	Cellular mechanism of ADPN	Cellular effect	General effect	Reference
Macrophages	AdipoR1 and AdipoR2 suppression (by M1 macrophages) or activation (by M2 macrophages)	In M1 macrophages, induce TNFα, IL-6 and IL-12 In M2 macrophages, induce IL-10	Pro- or anti-inflammatory depending on the polarization of the macrophages	van Stijn, C.M. et al., 2015 [35]
	38 MAPKs	28 of the 46 M1 markers (including TNFα and IL-6) and only 3 of the 43 M2 markers	Predominantly proinflammatory	Cheng, X. et al., 2012 [39]
	Nuclear translocation of FoxO3A and Atg5 and microtubule-associated protein light chain (LC3B), expression with ↑ formation of autophagosome	Inhibition of TNFα formation induced by LPS	Anti-inflammatory	Tilija, P.N. et al., 2015 [33]
	AMPK	↓ NFα, IL-6, mieloperoxidase, eotaxin	Anti-inflammatory	Zhu, L. et al., 2019 [169]
	AdipoR1	↑ IL-10, IL-1R and TIMP-1	Anti-inflammatory	Kumada, M. et al., 2004 [36]
	PPAR	Antioxidative effects	Reduction in the epithelial damage	Dellabianca, A. et al., 2020 [65]
	Binding to calreticulin	↑ Efferocytosis	Resolution in the inflammation	Takemura, Y. et el, 2007 [43]
	Adipo −/− mice with high resting NF, MM-12	↓ NFα, MM-12	Reduction in the inflammatory process	Summer, R. et al., 2008 [123]
Dendritic cells	AdipoR1 and AdipoR2?	PolarizationCD4+ lymphocytes towards Th1 and Th17 Induction of IL-12 and IL-1β/IL-23	Ag-specific Th1 and Th17 response	Jung, M.Y. et al., 2012 [170]
	AMPK-mTOR? PPAR?	↓ IL-12, Il-6, IL-10 expression of ↑ PDL-1 in T lymphocytes	Increase in Treg, limitation of the Th2 response	Tsang, J.Y. et al., 2011 [171]
ILC2	AMPK	↓ IL-33-NF-κB signaling	↓ Traffic of IL-33 from marrow	Wang, L. et al., 2021 [172]
Eosinophils	AdipoR1?	↓ in and eotaxin	↓ in eosinophils in the lung	Amarsaikhan, N. et al., 2019 [173]
Lymphocyte Th1	AMPK, mTOR Suppression of glycolysis independent of AMPK	↓ in IFNγ and IL17 ↓ glycolysis in T17 cells	↓ Th1-mediated response	Surendar, J. et al., 2019 [174]
	38 mitogen-activated protein kinases (MAPKs)	↑ IFNγ and IL-6	Proinflammatory	Cheng, X. et al., 2012 [39]
	Proapoptotic effect on influenza-activated cells	↓ in IFNγ, TNFα and IL-2 by influenza-activated cells	↓ Th1-mediated response	Wilk, S. et al., 2011 [175]
Lymphocyte Th2	AdipoR1, AdipoR2, T-cadherin?	Inhibition of IL-13, IL-5 release after sensitization with ovalbumin	↓ Th2 effects induced by allergen challenge	Shore, S.A. et al., 2006 [176]
Treg		↑ IL-10, IL-1Rα	Resolution of the inflammation	Ramos-Ramírez, P. et al., 2020 [177]

L = interleukin; MAPK= mitogen-activated protein kinase; TNFα = tumor necrosis factor α; AMPK = AMP-activated protein kinase; AdipoR = adiponectin receptor; TIMP-1 = tissue inhibitor of metalloproteinase 1; FoxO3A = a forkhead box O member of transcription factor; Atg = autophagy-protein 5; IL-1Rα = interleukin 1 receptor α; PPARα = peroxisome proliferator-activated receptor α; MM-12 = matrix metalloproteinase 12; Th = T helper lymphocytes; PDL-1 = programmed death-ligand 1; ILC = innate lymphoid cell; NF-κB = nuclear factor kappa-light-chain-enhancer of activated B cells; IFNγ = interferon γ; Treg = regulatory T cell. ↑= increase level; ↓= decrease level.

## References

[1] Singh S., Prakash Y.S., Linneberg A., Agrawal A. (2013). Insulin and the Lung: Connecting Asthma and Metabolic Syndrome. J. Allergy.

[2] Luo Y., Liu M. (2016). ADPN: A versatile player of innate immunity. J. Mol. Cell Biol..

[3] Scherer P.E., Williams S., Fogliano M., Baldini G., Lodish H.F. (1995). A novel serum protein similar to C1q; produced exclusively in adipocytes. J. Biol. Chem..

[4] Ye J.J., Bian X., Lim J., Medzhitov R. (2020). ADPN and related C1q/TNF-related proteins bind selectively to anionic phospholipids and sphingolipids. Proc. Natl. Acad. Sci. USA.

[5] Wang Y., Lam K.S.L., Yau M., Xu A. (2008). Post-translational modifications of ADPN: Mechanisms and functional implications. Biochem. J..

[6] Swarbrick M.M., Havel P.J. (2008). Physiological; pharmacological; and nutritional regulation of circulating ADPN concentrations in humans. Metab. Syndr. Relat. Disord..

[7] Qiang L., Wang H., Farmer S.R. (2007). ADPN secretion is regulated by SIRT1 and the endoplasmic reticulum oxidoreductase Ero1-L alpha. Mol. Cell Biol..

[8] Xie L., O’Reilly C.P., Chapes S.K., Mora S. (2008). ADPN and leptin are secreted through distinct trafficking pathways in adipocytes. Biochim. Biophys. Acta.

[9] Lim C.Y., Hong W., Han W. (2015). ADPN is released via a unique regulated exocytosis pathway from a pre-formed vesicle pool on insulin stimulation. Biochem. J..

[10] El Hachmane M.F., Komai A.M., Olofsson C.S. (2015). Cooling reduces cAMP-stimulated exocytosis and ADPN secretion at a Ca2+-dependent step in 3T3-L1 adipocytes. PLoS ONE.

[11] Musovic S., Olofsson C.S. (2019). Adrenergic stimulation of ADPN secretion in visceral mouse adipocytes is blunted in high-fat diet induced obesity. Sci. Rep..

[12] Komai A.M., Musovic S., Peris E., Alrifaiy A., El Hachmane M.F., Johansson M., Wernstedt Asterholm I., Olofsson C.S. (2016). White Adipocyte ADPN Exocytosis Is Stimulated via β3-Adrenergic Signaling and Activation of Epac1: Catecholamine Resistance in Obesity and Type 2 Diabetes. Diabetes.

[13] Kovacova Z., Vitkova M., Kovacikova M., Klimcakova E., Bajzova M., Hnevkovska Z., Rossmeislova L., Stich V., Langin D., Polak J. (2009). Secretion of ADPN multimeric complexes from adipose tissue explants is not modified by very low calorie diet. Eur. J. Endocrinol..

[14] Guerre-Millo M. (2008). ADPN: An update. Diabetes Metab..

[15] Fasshauer M., Kralisch S., Klier M., Lossner U., Bluher M., Klein J., Paschke R. (2003). ADPN gene expression and secretion is inhibited by interleukin-6 in 3T3-L1 adipocytes. Biochem. Biophys. Res. Commun..

[16] Yuan G., Chen X., Ma Q., Qiao J., Li R., Li X., Li S., Tang J., Zhou L., Song H. (2007). C-reactive protein inhibits ADPN gene expression and secretion in 3T3-L1 adipocytes. J. Endocrinol..

[17] Juan C.C., Chuang T.Y., Chang C.L., Huang S.W., Ho L.T. (2007). Endothelin-1 Regulates ADPN Gene Expression and Secretion in 3T3-L1 Adipocytes via Distinct Signaling Pathways. Endocrinology.

[18] Yamauchi T., Kamon J., Minokoshi Y., Ito Y., Waki H., Uchida S., Yamashita S., Noda M., Kita S., Ueki K. (2002). ADPN stimulates glucose utilization and fatty-acid oxidation by activating AMP-activated protein kinase. Nat. Med..

[19] Fruebis J., Tsao T.S., Javorschi S., Ebbets-Reed D., Erickson M.R., Yen F.T., Bihain B.E., Lodish H.F. (2001). Proteolytic cleavage product of 30-kDa adipocyte complement-related protein increases fatty acid oxidation in muscle and causes weight loss in mice. Proc. Natl. Acad. Sci. USA.

[20] Combs T.P., Berg A.H., Obici S., Scherer P.E., Rossetti L. (2001). Endogenous glucose production is inhibited by the adipose-derived protein Acrp30. J. Clin. Investig..

[21] Tomas E., Tsao T.S., Saha A.K., Murrey H.E., Zhang C.C., Itani S.I., Lodish H.F., Ruderman N.B. (2002). Enhanced muscle fat oxidation and glucose transport by ACRP30 globular domain: Acetyl-CoA carboxylase inhibition and AMP-activated protein kinase activation. Proc. Natl. Acad. Sci. USA.

[22] Krause M.P., Milne K.J., Hawke T.J. (2019). ADPN-Consideration for its Role in Skeletal Muscle Health. Int. J. Mol. Sci..

[23] Qi Y., Takahashi N., Hileman S., Patel H.R., Berg A.H., Pajvani U.B., Scherer P.E., Ahima R.S. (2004). ADPN acts in the brain to decrease body weight. Nat. Med..

[24] Kubota N., Yano W., Kubota T., Yamauchi T., Itoh S., Kumagai H., Kozono H., Takamoto I., Okamoto S., Shiuchi T. (2007). ADPN stimulates AMP-activated protein kinase in the hypothalamus and increases food intake. Cell Metab..

[25] Wheatley K.E., Nogueira L.M., Perkins S.N., Hursting S.D. (2011). Differential effects of calorie restriction and exercise on the adipose transcriptome in diet-induced obese mice. J. Obes..

[26] Liu Y.M., Lacorte J.M., Viguerie N., Poitou C., Pelloux V., Guy-Grand B., Coussieu C., Langin D., Basdevant A., Clément K. (2003). ADPN gene expression in subcutaneous adipose tissue of obese women in response to short-term very low calorie diet and refeeding. J. Clin. Endocrinol. Metab..

[27] Lee B., Shao J. (2014). ADPN and energy homeostasis. Rev. Endocr. Metab. Disord..

[28] 28Ajuwon K.M., Spurlock M.E. (2005). ADPN inhibits LPS-induced NF-kappaB activation and IL-6 production and increases PPARgamma2 expression in adipocytes. Am. J. Physiol. Regul. Integr. Comp. Physiol..

[29] Okamoto Y., Folco E.J., Minami M., Wara A.K.M.K., Feinberg M.W., Sukhova G.K., Colvin R.A., Kihara S., Funahashi T., Luster A.D. (2008). ADPN inhibits the production of CXC receptor 3 chemokine ligands in macrophages and reduces T-lymphocyte recruitment in atherogenesis. Circ. Res..

[30] Fayad R., Pini M., Sennello J.A., Cabay R.J., Chan L., Xu A., Fantuzzi G. (2007). ADPN deficiency protects mice from chemically induced colonic inflammation. Gastroenterology.

[31] Tang C.H., Chiu Y.C., Tan T.W., Yang R.S., Fu W.M. (2007). ADPN enhances IL-6 production in human synovial fibroblast via an AdipoR1 receptor; AMPK; p38; and NF-kappa B pathway. J. Immunol..

[32] Lovren F., Pan Y., Quan A., Szmitko P.E., Singh K.K., Shukla P.C., Gupta M., Chan L., Al-Omran M., Teoh H. (2010). ADPN primes human monocytes into alternative anti-inflammatory M2 macrophages. Am. J. Physiol. Heart Circ. Physiol..

[33] Tilija P.N., Subedi A., Kim M.J., Park P.-H. (2015). Globular ADPN Causes Tolerance to LPS-Induced TNF-α Expression via Autophagy Induction in RAW 264.7 Macrophages: Involvement of SIRT1/FoxO3A Axis. PLoS ONE.

[34] Luo N., Chung B.H., Wang X., Klein K.L., Tang C.K., Garvey W.T., Fua Y. (2013). Enhanced ADPN actions by overexpression of ADPN receptor 1 in macrophages. Atherosclerosis.

[35] van Stijn C.M., Kim J., Lusis A.J., Barish G.D., Tangirala R.K. (2015). Macrophage polarization phenotype regulates ADPN receptor expression and ADPN anti-inflammatory response. FASEB J..

[36] Kumada M., Kihara S., Ouchi N., Kobayashi H., Okamoto Y., Ohashi K., Maeda K., Nagaretani H., Kishida K., Maeda N. (2004). ADPN specifically increased tissue inhibitor of metalloproteinase-1 through interleukin-10 expression in human macrophages. Circulation.

[37] Wolf A.M., Wolf D., Rumpold H., Enrich B., Tilg H. (2004). ADPN induces the anti-inflammatory cytokines IL-10 and IL-1RA in human leukocytes. Biochem. Biophys. Res. Commun..

[38] Ramos-Ramírez P., Malmhäll C., Tliba O., Rådinger M., Bossios A. (2021). ADPN/AdipoR1 Axis Promotes IL-10 Release by Human Regulatory T Cells. Front. Immunol..

[39] Cheng X., Folco E.J., Shimizu K., Libby P. (2012). ADPN induces pro-inflammatory programs in human macrophages and CD4+ T cells. J. Biol. Chem..

[40] Tsatsanis C., Zacharioudaki V., Androulidaki A., Dermitzaki E., Charalampopoulos I., Minas V., Gravanis A., Margioris A.N. (2005). ADPN induces TNF-alpha and IL-6 in macrophages and promotes tolerance to itself and other pro-inflammatory stimuli. Biochem. Biophys. Res. Commun..

[41] Hug C., Wang J., Ahmad N.S., Bogan J.S., Tsao T.S., Lodish H.F. (2004). T-cadherin is a receptor for hexameric and high-molecular-weight forms of Acrp30/ADPN. Proc. Natl. Acad. Sci. USA.

[42] Williams A.S., Kasahara D.I., Verbout N.G., Fedulov A.V., Zhu M., Si H., Wurmbrand A.P., Hug C., Ranscht B., Shore S.A. (2012). Role of the ADPN binding protein; T-cadherin (Cdh13); in allergic airways responses in mice. PLoS ONE.

[43] Takemura Y., Ouchi N., Shibata R., Aprahamian T., Kirber M.T., Summer R.S., Kihara S., Walsh K. (2007). ADPN modulates inflammatory reactions via calreticulin receptor-dependent clearance of early apoptotic bodies. J. Clin. Investig..

[44] Sternberg J., Wankell M., Subramaniam V.N., Hebbard L.W. (2017). The functional roles of T-cadherin in mammalian biology. AIMS Molec. Sci..

[45] Parker-Duffen J.L., Nakamura K., Silver M., Kikuchi R., Tigges U., Yoshida S., Denzel M.S., Ranscht B., Walsh K. (2013). T-cadherin is essential for ADPN mediated revascularization. J. Biol. Chem.

[46] Ouchi N., Walsh K. (2007). ADPN as an anti-inflammatory factor. Clin. Chim. Acta.

[47] Steinberg G.R., Carling D. (2019). AMP-activated protein kinase: The current landscape for drug development. Nat. Rev. Drug Discov..

[48] Mancini S.J., White A.D., Bijland S., Rutherford C., Graham D., Richter E.A., Viollet B., Touyz R.M., Palmer T.M., Salt I.P. (2017). Activation of AMP-activated protein kinase rapidly suppresses multiple pro-inflammatory pathways in adipocytes including IL-1 receptor-associated kinase-4 phosphorylation. Mol. Cell Endocrinol..

[49] Michalek R.D., Gerriets V.A., Jacobs S.R., Macintyre A.N., MacIver N.J., Mason E.F., Sullivan S.A., Nichols A.G., Rathmell J.C. (2011). Cutting edge: Distinct glycolytic and lipid oxidative metabolic programs are essential for effector and regulatory CD4+ T cell subsets. J. Immunol..

[50] Li D., Song L., Wang J., Meng C., Cui X. (2018). ADPN protects against lung ischemia-reperfusion injury in rats with type 2 diabetes mellitus. Mol. Med. Rep..

[51] Yuan F., Li Y.N., Liu Y.H., Yi B., Tian J.W., Liu F.Y. (2012). ADPN inhibits the generation of reactive oxygen species induced by high glucose and promotes endothelial NO synthase formation in human mesangial cells. Mol. Med. Rep..

[52] Wang F., Liu Y., Yang W., Yuan J., Mo Z. (2018). ADPN inhibits NLRP3 inflammasome by modulating the AMPK-ROS pathway. Int. J. Clin. Exp. Pathol..

[53] Li R., Wang W.Q., Zhang H., Yang X., Fan Q., Christopher T.A., Lopez B.L., Tao L., Goldstein B.J., Gao F. (2007). ADPN improves endothelial function in hyperlipidemic rats by reducing oxidative/nitrative stress and differential regulation of eNOS/iNOS activity. Am. J. Physiol. Endocrinol. Metab..

[54] Xin X., Zhou L., Reyes C.M., Liu F., Dong L.Q. (2011). APPL1 mediates ADPN-stimulated p38 MAPK activation by scaffolding the TAK1-MKK3-p38 MAPK pathway. Am. J. Physiol. Endocrinol. Metab..

[55] Cuenda A., Rousseau S. (2007). p38 MAP-kinases pathway regulation; function and role in human diseases. Biochim. Biophys. Acta.

[56] Yoon M.J., Lee G.Y., Chung J.J., Ahn Y.H., Hong S.H., Kim J.B. (2006). ADPN increases fatty acid oxidation in skeletal muscle cells by sequential activation of AMP-activated protein kinase; p38 mitogen-activated protein kinase; and peroxisome proliferator-activated receptor alpha. Diabetes.

[57] Yamauchi T., Iwabu M., Okada-Iwabu M., Kadowaki T. (2014). ADPN receptors: A review of their structure; function and how they work. Best Pract. Res. Clin. Endocrinol. Metab..

[58] Heiker J.T., Kosel D., Beck-Sickinger A.G. (2010). Molecular mechanisms of signal transduction via ADPN and ADPN receptors. Biol. Chem..

[59] Devchand P.R., Keller H., Peters J.M., Vazquez M., Gonzalez F.J., Wahli W. (1996). The PPARalpha-leukotriene B4 pathway to inflammation control. Nature.

[60] Narala V.R., Adapala R.K., Suresh M.V., Brock T.G., Peters-Golden M., Reddy R.C. (2010). Leukotriene B4 is a physiologically relevant endogenous peroxisome proliferator-activated receptor-alpha agonist. J. Biol. Chem..

[61] Koeberle A., Siemoneit U., Northoff H., Hofmann B., Schneider G., Werz O. (2009). MK-886; an inhibitor of the 5-lipoxygenase-activating protein; inhibits cyclooxygenase-1 activity and suppresses platelet ag-gregation. Eur. J. Pharmacol..

[62] Delayre-Orthez C., Becker J., Auwerx J., Frossard N., Pons F. (2008). Suppression of allergen-induced airway inflammation and immune response by the peroxisome proliferator-activated receptor-alpha agonist fenofibrate. Eur. J. Pharmacol..

[63] Elaidy S.M., Essawy S.S., Hussain M.A., El-Kherbetawy M.K., Hamed E.R. (2018). Modulation of the IL-23/IL-17 axis by fenofibrate ameliorates the ovalbumin/lipopolysaccharide-induced airway inflammation and bronchial asthma in rats. Naunyn. Schmiedebergs Arch. Pharmacol..

[64] Bougarne N., Weyers B., Desmet S.J., Deckers J., Ray D.W., Staels B., De Bosscher K. (2018). Molecular Actions of PPARα in Lipid Metabolism and Inflammation. Endocr. Rev..

[65] Dellabianca A., Beretta C., Faniglione M., De Angelis S., Colucci M., Cervio M., Tonini S., Candura S.M. (2020). A PPAR-α agonist protects the non-adrenergic; non-cholinergic inhibitory system of guinea pig trachea from the effect of inhaled ammonium persulphate: A pilot study. G Ital. Med. Lav. Ergon..

[66] Okayasu T., Tomizawa A., Suzuki K., Manaka K., Hattori Y. (2008). PPARalpha activators upregulate eNOS activity and inhibit cytokine-induced NF-kappaB activation through AMP-activated protein kinase activation. Life Sci..

[67] Vargas-Sánchez K., Vargas L., Urrutia Y., Beltrán I., Rossi A.B., Lozano H.Y., Guarín J., Losada-Barragán M. (2020). PPARα and PPARβ/δ are negatively correlated with proinflammatory markers in leukocytes of an obese pediatric population. J. Inflamm..

[68] Hwang S.J., Kim J.H., Shim J.W., Kim D.S., Jung H.L., Park M.S., Lee W.Y., Kim S.Y., Shim J.Y. (2011). Peroxisome proliferator-activated receptor-gamma expression in the lung tissue of obese rats. Yonsei Med. J..

[69] Sugii S., Olson P., Sears D.D., Saberi M., Atkins A.R., Barish G.D., Hong S.H., Castro G.L., Yin Y.Q., Nelson M.C. (2009). PPARgamma activation in adipocytes is sufficient for systemic insulin sensitization. Proc. Natl. Acad. Sci. USA.

[70] Shen W., Hao J., Feng Z., Tian C., Chen W., Packer L., Shi X., Zang W., Liu J. (2011). Lipoamide or lipoic acid stimulates mitochondrial biogenesis in 3T3-L1 adipocytes via the endothelial NO synthase-cGMP-protein kinase G signalling pathway. Br. J. Pharmacol..

[71] Hernandez-Quiles M., Broekema M.F., Kalkhoven E. (2021). PPARgamma in Metabolism; Immunity; and Cancer: Unified and Diverse Mechanisms of Action. Front. Endocrinol..

[72] Astapova O., Leff T. (2012). ADPN and PPARγ: Cooperative and interdependent actions of two key regulators of metabolism. Vitam. Horm..

[73] Koenen T.B., Tack C.J., Kroese J.M., Hermus A.R., Sweep F.C.G., van der Laak J., Stalenhoef A.F., de Graaf J., van Tits L.J., Stienstra R. (2009). Pioglitazone treatment enlarges subcutaneous adipocytes in insulin-resistant patients. J. Clin. Endocrinol. Metab..

[74] Kita A., Yamasaki H., Kuwahara H., Moriuchi A., Fukushima K., Kobayashi M., Fukushima T., Takahashi R., Abiru N., Uotani S. (2005). Identification of the promoter region required for human ADPN gene transcription: Association with CCAAT/enhancer binding protein-beta and tumor necrosis factor-alpha. Biochem. Biophys. Res. Commum..

[75] Long Q., Lei T., Feng B., Yin C., Jin D., Wu Y., Zhu X., Chen X., Gan L., Yang Z. (2010). Peroxisome proliferatoractivated receptor-gamma increases ADPN secretion via transcriptional repression of endoplasmic reticulum chaperone protein ERp44. Endocrinology.

[76] Riera-Guardia N., Rothenbacher D. (2008). The effect of thiazolidinediones on ADPN serum level: A meta-analysis. Diabetes Obes. Metab..

[77] Shin N.R., Park S.H., Ko J.W., Cho Y.K., Lee I.C., Kim J.C., Shin I.S., Kim J.S. (2018). Lobeglitazone Attenuates Airway Inflammation and Mucus Hypersecretion in a Murine Model of Ovalbumin-Induced Asthma. Front. Pharmacol..

[78] Halberg N., Schraw T.D., Wang Z.V., Kim J.Y., Yi J., Hamilton M.P., Luby-Phelps K., Scherer P.E. (2009). Systemic fate of the adipocyte-derived factor ADPN. Diabetes.

[79] Shimotomai T., Kakei M., Narita T., Koshimura J., Hosoba M., Kato M., Komatsuda A., Ito S. (2005). Enhanced urinary ADPN excretion in IgA-nephropathy patients with proteinuria. Ren. Fail..

[80] Heidari M., Nasri P., Nasri H. (2015). ADPN and chronic kidney disease; a review on recent findings. J. Nephropharmacol..

[81] Lausten-Thomsen U., Christiansen M., Fonvig C.E., Trier C., Pedersen O., Hansen T., Holm J.C. (2015). Reference values for serum total ADPN in healthy non-obese children and adolescents. Clin. Chim. Acta.

[82] Cnop M., Havel P.J., Utzschneider K.M., Carr D.B., Sinha M.K., Boyko E.J., Retzlaff B.M., Knopp R.H., Brunzell J.D., Kahn S.E. (2003). Relationship of ADPN to body fat distribution; insulin sensitivity and plasma lipoproteins: Evidence for independent roles of age and sex. Diabetologia.

[83] Nien J.K., Mazaki-Tovi S., Romero R., Erez O., Kusanovic J.P., Gotsch F., Pineles B.L., Gomez R., Edwin S., Mazor M. (2007). Plasma ADPN concentrations in non-pregnant; normal and overweight pregnant women. J. Perinat. Med..

[84] Gavrila A., Chan J.L., Yiannakouris N., Kontogianni M., Miller L.C., Orlova C., Mantzoros C.S. (2003). Serum ADPN Levels Are Inversely Associated with Overall and Central Fat Distribution but Are Not Directly Regulated by Acute Fasting or Leptin Administration in Humans: Cross-Sectional and Interventional Studies. J. Clin. Endocrinol. Metab..

[85] Silva-Nunes J., Oliveira A., Duarte L., Barradas M., Melão A., Brito M., Veiga L. (2013). Factors related with adiponectinemia in obese and normalweight women and with its variation in weight loss programs. Obes. Facts.

[86] Salem A.M., Latif R., Rafique N. (2019). Comparison of ADPN Levels During the Menstrual Cycle Between Normal Weight and Overweight/Obese Young Females. Physiol. Res..

[87] Wang H., Wu J., Gu W., Wang B., Wan F., Dai B., Zhang H., Shi G., Shen Y., Zhu Y. (2016). Serum ADPN Level May be an Independent Predictor of Clear Cell Renal Cell Carcinoma. J. Cancer..

[88] Allison M.A., Criqui M.H., Maisel A.S., Daniels L.B., Roberts C.K., Polak J.F., Cushman M. (2015). ADPN is independently associated with NT-proBNP: The Multi-Ethnic Study of Atherosclerosis. Nutr. Metab. Cardiovasc. Dis..

[89] Wannamethee S.G., Welsh P., Whincup P.H., Sawar N., Thomas M.C., Gudnarsson V., Sattar N. (2011). High ADPN and increased risk of cardiovascular disease and mortality in asymptomatic older men: Does NT-proBNP help to explain this association?. Eur. J. Cardiovasc. Prev. Rehabil..

[90] Rutkowski J.M., Pastor J., Sun K., Park S.K., Bobulescu I.A., Chen C.T., Moe O.W., Scherer P.E. (2017). ADPN alters renal calcium and phosphate excretion through regulation of klotho expression. Kidney Int..

[91] Komici K., Iacono A.D., De Luca A., Perrotta F., Bencivenga L., Rengo G., Rocca A., Guerra G. (2021). ADPN and Sarcopenia: A Systematic Review with Meta-Analysis. Front. Endocrinol..

[92] Li S., Shin H.J., Ding E.L., van Dam R.M. (2009). ADPN Levels and Risk of Type 2 Diabetes: A Systematic Review and Meta-analysis. JAMA.

[93] Sook Lee E., Park S.S., Kim E., Sook Yoon Y., Ahn H.Y., Park C.Y., Ho Yun Y., Woo Oh S. (2013). Association between ADPN levels and coronary heart disease and mortality: A systematic review and meta-analysis. Int. J. Epidemiol..

[94] Menon V., Li L., Wang X., Greene T., Balakrishnan V., Madero M., Pereira A.A., Beck G.J., Kusek J.W., Collins A.J. (2006). ADPN and mortality in patients with chronic kidney disease. J. Am. Soc. Nephrol..

[95] Borges M.C., Barros A.J.D., Ferreira D.L.S., Casas J.P., Horta B.L., Kivimaki M., Kumari M., Menon U., Gaunt T.R., Ben-Shlomo Y. (2017). Metabolic Profiling of ADPN Levels in Adults: Mendelian Randomization Analysis. Circ. Cardiovasc. Genet..

[96] Sood A., Qualls C., Schuyler M., Thyagarajan B., Steffes M.W., Smith L.J., Jacobs D.R. (2012). Low serum ADPN predicts future risk for asthma in women. Am. J. Respir. Crit Care Med..

[97] Barton J.H., Ireland A., Fitzpatrick M., Kessinger C., Camp D., Weinman R., McMahon D., Leader J.K., Holguin F., Wenzel S.E. (2016). Adiposity influences airway wall thickness and the asthma phenotype of HIV-associated obstructive lung disease: A cross-sectional study. BMC Pulm. Med..

[98] Lu Y., Andiappan A.K., Lee B., Ho R., Lim T.K., Kuan W.S., Goh D.Y., Mahadevan M., Sim T.B., Wang Y. (2016). Neuropeptide Y associated with asthma in young adults. Neuropeptides.

[99] Capelo A.V., da Fonseca V.M., Peixoto M.V., de Carvalho S.R., Azevedo C.M., Elsas M.I., Marques B. (2006). Visceral adiposity is associated with cytokines and decrease in lung function in women with persistent asthma. Rev. Port. Pneumol..

[100] Magrone T., Simone M., Altamura M., Munno I. (2014). Characterization of the immune inflammatory profile in obese asthmatic children. Endocr. Metab. Immune Disord. Drug Targets.

[101] Aydin M., Koca C., Ozol D., Uysal S., Yildirim Z., Kavakli H.S., Yigitoglu M.R. (2013). Interaction of metabolic syndrome with asthma in postmenopausal women: Role of adipokines. Inflammation.

[102] Jensen M.E., Gibson P.G., Collins C.E., Wood L.G. (2013). Airway and systemic inflammation in obese children with asthma. Eur. Respir. J..

[103] Carpio C., Villasante C., Galera R., Romero D., de Cos A., Hernanz A., García-Río F. (2016). Systemic inflammation and higher perception of dyspnea mimicking asthma in obese subjects. J. Allergy Clin. Immunol..

[104] Ozde C., Dogru M., Erdogan F., Ipek I.O., Ozde S., Karakaya O. (2015). The relationship between adiponectin levels and epicardial adipose tissue thickness in non-obese children with asthma. Asian Pac. J. Allergy Immunol..

[105] Lu Y., Van Bever H.P., Lim T.K., Kuan W.S., Goh D.Y., Mahadevan M., Sim T.B., Ho R., Larbi A., Ng T.P. (2015). Obesity, asthma prevalence and IL-4: Roles of inflammatory cytokines, adiponectin and neuropeptide Y. Pediatr. Allergy Immunol..

[106] Baltieri L., Cazzo E., de Souza A.L., Alegre S.M., de Paula Vieira R., Antunes E., de Mello G.C., Claudio Martins L., Chaim E.A. (2018). Influence of weight loss on pulmonary function and levels of adipokines among asthmatic individuals with obesity: One-year follow-up. Respir. Med..

[107] van Huisstede A., Rudolphus A., Castro Cabezas M., Biter L.U., van de Geijn G.J., Taube C., Hiemstra P.S., Braunstahl G.J. (2015). Effect of bariatric surgery on asthma control, lung function and bronchial and systemic inflammation in morbidly obese subjects with asthma. Thorax.

[108] Sideleva O., Suratt B.T., Black K.E., Tharp W.G., Pratley R.E., Forgione P., Dienz O., Irvin C.G., Dixon A.E. (2012). Obesity and asthma: An inflammatory disease of adipose tissue not the airway. Am. J. Respir. Crit. Care Med..

[109] Leão da Silva P., de Mello M.T., Cheik N.C., Sanches P.L., Munhoz da Silveira Campos R., Carnier J., Inoue D., do Nascimento C.M., Oyama L.M., Tock L. (2012). Reduction in the leptin concentration as a predictor of improvement in lung function in obese adolescents. Obes. Facts.

[110] Abd El-Kader M.S., Al-Jiffri O., Ashmawy E.M. (2013). Impact of weight loss on markers of systemic inflammation in obese Saudi children with asthma. Afr. Health Sci..

[111] Tsaroucha A., Daniil Z., Malli F., Georgoulias P., Minas M., Kostikas K., Bargiota A., Zintzaras E., Gourgoulianis K.I. (2013). Leptin, adiponectin, and ghrelin levels in female patients with asthma during stable and exacerbation periods. J. Asthma..

[112] Mikalsen I.B., Byberg K., Forman M.R., Øymar K. (2020). Adipokines in adolescence; the associations with lung function and atopy—A cross-sectional study. Respir. Med..

[113] Huang F., Del-Río-Navarro B.E., Torres-Alcántara S., Pérez-Ontiveros J.A., Ruiz-Bedolla E., Saucedo-Ramírez O.J., Villafaña S., Sánchez Muñoz F., Bravo G., Hong E. (2017). Adipokines, asymmetrical dimethylarginine, and pulmonary function in adolescents with asthma and obesity. J. Asthma..

[114] Grotta M.B., Squebola-Cola D.M., Toro A.A., Ribeiro M.A., Mazon S.B., Ribeiro J.D., Antunes E. (2013). Obesity increases eosinophil activity in asthmatic children and adolescents. BMC Pulm. Med..

[115] Song W.J., Kim S.H., Lim S., Park Y.J., Kim M.H., Lee S.M., Lee S.B., Kim K.W., Jang H.C., Cho S.H. (2012). Association between obesity and asthma in the elderly population: Potential roles of abdominal subcutaneous adiposity and sarcopenia. Ann. Allergy Asthma Immunol..

[116] Matsumoto Y., Toyomasu K., Uchimura N., Ishitake T. (2013). Low-molecular-weight adiponectin is more closely associated with episodes of asthma than high-molecular-weight ADPN. Endocr. J..

[117] Hayashikawa Y., Iwata M., Inomata M., Kawagishi Y., Tokui K., Taka C., Kambara K., Okazawa S., Yamada T., Hayashi R. (2015). Association of serum adiponectin with asthma and pulmonary function in the Japanese population. Endocr. J..

[118] Ma C., Wang Y., Xue M. (2019). Correlations of severity of asthma in children with body mass index, adiponectin and leptin. J. Clin. Lab. Anal..

[119] Ceylan E., Bulut S., Yilmaz M., Örün H., Karadağ F., Ömürlü İ.K., Kirdar S., Karul A. (2019). The Levels of Serum Biomarkers in Stable Asthma Patients with Comorbidities. Iran. J. Allergy Asthma Immunol..

[120] Kalmarzi R.N., Ataee P., Mansori M., Moradi G., Ahmadim S., Kaviani Z., Khalafi B., Kooti W. (2017). Serum levels of adiponectin and leptin in asthmatic patients and its relation with asthma severity, lung function and BMI. Allergol. Immunopathol..

[121] Zhang X., Zheng J., Zhang L., Liu Y., Chen G.P., Zhang H.P., Wang L., Kang Y., Wood L.G., Wang G. (2018). Systemic inflammation mediates the detrimental effects of obesity on asthma control. Allergy Asthma Proc..

[122] Ballantyne D., Scott H., MacDonald-Wicks L., Gibson P.G., Wood L.G. (2016). Resistin is a predictor of asthma risk and resistin:adiponectin ratio is a negative predictor of lung function in asthma. Clin. Exp. Allergy.

[123] Ding Y., Yang H., He H., Shi H., He P., Yan S., Jin T. (2015). Plasma Adiponectin Concentrations and Adiponectin Gene Polymorphisms Are Associated with Bronchial Asthma in the Chinese Li Population. Iran. J. Allergy Asthma Immunol..

[124] de Lima Azambuja R., da Costa Santos Azambuja L.S., Costa C., Rufino R. (2015). Adiponectin in Asthma and Obesity: Protective Agent or Risk Factor for More Severe Disease?. Lung.

[125] Dogru M., Ozde S., Aktas A., Yuksel Karatoprak E. (2015). The Adiponectin levels and asthma control in non-obese children with asthma. J. Asthma.

[126] Newson R.B., Jones M., Forsberg B., Janson C., Bossios A., Dahlen S.E., Toskala E.M., Al-Kalemji A., Kowalski M.L., Rymarczyk B. (2014). The association of asthma, nasal allergies, and positive skin prick tests with obesity, leptin, and adiponectin. Clin. Exp. Allergy.

[127] Biagioni B.J., Pui M.M., Fung E., Wong S., Hosseini A., Dybuncio A., Alexis N.E., Carlsten C. (2014). Sputum adiponectin as a marker for western red cedar asthma. J. Allergy Clin. Immunol..

[128] Sood A., Seagrave J., Herbert G., Harkins M., Alam Y., Chiavaroli A., Shohreh R., Montuschi P., Campen M., Harmon M. (2014). High sputum total adiponectin is associated with low odds for asthma. J. Asthma..

[129] Dorevitch S., Conroy L., Karadkhele A., Rosul L., Stacewicz-Sapuntzakis M., Fantuzzi G. (2013). Associations between obesity and asthma in a low-income, urban, minority population. Ann. Allergy Asthma Immunol..

[130] Zhu X.L., Qin X.Q., Xiang Y., Tan Y.R., Qu X.P., Liu H.J. (2013). Adipokine ADPN is a potential protector to human bronchial epithelial cell for regulating proliferation, wound repair and apoptosis: Comparison with leptin and resistin. Peptides.

[131] Salvator H., Grassin-Delyle S., Naline E., Brollo M., Fournier C., Couderc L.J., Devillier P. (2020). Contrasting Effects of Adipokines on the Cytokine Production by Primary Human Bronchial Epithelial Cells: Inhibitory Effects of ADPN. Front. Pharmacol..

[132] Wang Y., Wang X., Lau W.B., Yuan Y., Booth D., Li J.J., Scalia R., Preston K., Gao E., Koch W. (2014). ADPN inhibits tumor necrosis factor-α-induced vascular inflammatory response via caveolin-mediated ceramidase recruitment and activation. Circ. Res..

[133] Summer R., Little F.F., Ouchi N., Takemura Y., Aprahamian T., Dwyer D., Fitzsimmons K., Suki B., Parameswaran H., Fine A. (2008). Alveolar macrophage activation and an emphysema-like phenotype in ADPN-deficient mice. Am. J. Physiol. Lung Cell Mol. Physiol..

[134] Mukhopadhyay S., Sypek J., Tavendale R., Gartner U., Winter J., Li W., Page K., Fleming M., Brady J., O’Toole M. (2010). Matrix metalloproteinase-12 is a therapeutic target for asthma in children and young adults. J. Allergy Clin. Immunol..

[135] Vandenbroucke R.E., Dejonckheere E., Libert C. (2011). A therapeutic role for matrix metalloproteinase inhibitors in lung diseases?. Eur. Respir. J..

[136] Zappalà G., Rechler M.M. (2009). IGFBP-3, hypoxia and TNF-alpha inhibit ADPN transcription. Biochem. Biophys. Res. Commun..

[137] He Y., Lu L., Wei X., Jin D., Qian T., Yu A., Sun J., Cui J., Yang Z. (2016). The multimerization and secretion of ADPN are regulated by TNF-alpha. Endocrine.

[138] Itoh M., Suganami T., Satoh N., Tanimoto-Koyama K., Yuan X., Tanaka M., Kawano H., Yano T., Aoe S., Takeya M. (2007). Increased ADPN secretion by highly purified eicosapentaenoic acid in rodent models of obesity and human obese subjects. Arterioscler. Thromb. Vasc. Biol..

[139] Suganami T., Nishida J., Ogawa Y. (2005). A paracrine loop between adipocytes and macrophages aggravates inflammatory changes: Role of free fatty acids and tumor necrosis factor α. Arterioscler. Thromb. Vasc. Biol..

[140] Chambrier C., Bastard J.P., Rieusset J., Chevillotte E., Bonne-font-Rousselot D., Therond P., Hainque B., Riou J.P., Laville M., Vidal H. (2002). Eicosapentaenoic acid induces mRNA expression of peroxisome proliferator-activated receptor gamma. Obes. Res..

[141] Zheng X.Y., Orellano P., Lin H.L., Jiang M., Guan W.J. (2021). Short-term exposure to ozone, nitrogen dioxide, and sulphur dioxide and emergency department visits and hospital admissions due to asthma: A systematic review and meta-analysis. Environ. Int..

[142] Kasahara D.I., Kim H.Y., Williams A.S., Verbout N.G., Tran J., Si H., Wurmbrand A.P., Jastrab J., Hug C., Umetsu D.T. (2012). Pulmonary inflammation induced by subacute ozone is augmented in ADPN-deficient mice: Role of IL-17A. J. Immunol..

[143] Ozier A., Allard B., Bara I., Girodet P.O., Trian T., Marthan R., Berger P. (2011). The pivotal role of airway smooth muscle in asthma pathophysiology. J. Allergy.

[144] Mahn K., Hirst S.J., Ying S., Holt M.R., Lavender P., Ojo O.O., Siew L., Simcock D.E., McVicker C.G., Kanabar V. (2009). Diminished sarco/endoplasmic reticulum Ca2+ ATPase (SERCA) expression contributes to airway remodelling in bronchial asthma. Proc. Natl. Acad. Sci. USA.

[145] Guo J., Bian Y., Bai R., Li H., Fu M., Xiao C. (2013). Globular ADPN attenuates myocardial ischemia/reperfusion injury by upregulating endoplasmic reticulum Ca^2^⁺-ATPase activity and inhibiting endoplasmic reticulum stress. J. Cardiovasc. Pharmacol..

[146] Manson M.L., Säfholm J., James A., Johnsson A.K., Bergman P., Al-Ameri M., Orre A.C., Kärrman-Mårdh C., Dahlén S.E., Adner M. (2020). IL-13 and IL-4, but not IL-5 nor IL-17A, induce hyperresponsiveness in isolated human small airways. J. Allergy Clin. Immunol..

[147] Moynihan B., Tolloczko B., Michoud M.C., Tamaoka M., Ferraro P., Martin J.G. (2008). MAP kinases mediate interleukin-13 effects on calcium signaling in human airway smooth muscle cells. Am. J. Physiol. Lung Cell Mol. Physiol..

[148] Verbout N.G., Benedito L., Williams A.S., Kasahara D.I., Wurmbrand A.P., Si H., Halayko A.J., Hug C., Shore S.A. (2013). Impact of ADPN overexpression on allergic airways responses in mice. J. Allergy (Cairo).

[149] Kerr S.C., Carrington S.D., Oscarson S., Gallagher M.E., Solon M., Yuan S., Ahn J.N., Dougherty R.H., Finkbeiner W.E., Peters M.C. (2014). Intelectin-1 is a prominent protein constituent of pathologic mucus associated with eosinophilic airway inflammation in asthma. Am. J. Respir. Crit Care Med..

[150] Bonser L.R., Erle D.J. (2017). Airway Mucus and Asthma: The Role of MUC5AC and MUC5B. J. Clin. Med..

[151] Chu H.W., Balzar S., Westcott J.Y., Trudeau J.B., Sunm Y., Conrad D.J., Wenzel S.E. (2002). Expression and activation of 15-lipoxygenase pathway in severe asthma: Relationship to eosinophilic phenotype and collagen deposition. Clin. Exp. Allergy.

[152] Watanabe T., Chibana K., Shiobara T., Tei R., Koike R., Nakamura Y., Arai R., Horigane Y., Shimizu Y., Takemasa A. (2017). Expression of intelectin-1 in bronchial epithelial cells of asthma is correlated with T-helper 2 (Type-2) related parameters and its function. Allergy Asthma Clin. Immunol..

[153] Mashima R., Okuyama T. (2015). The role of lipoxygenases in pathophysiology; new insights and future perspectives. Redox Biol..

[154] James A.L., Noble P.B., Drew S.A., Mauad T., Bai T.R., Abramson M.J., McKay K.O., Green F.H.Y., Elliot J.G. (2018). Airway smooth muscle proliferation and inflammation in asthma. J. Appl. Physiol..

[155] Liu L., Pan Y., Song Y., Su X., Ke R., Yang L., Gao L., Li M. (2016). Activation of AMPK α2 inhibits airway smooth muscle cells proliferation. Eur. J. Pharmacol..

[156] Pan Y., Liu L., Li S., Wang K., Ke R., Shi W., Wang J., Yan X., Zhang Q., Wang Q. (2018). Activation of AMPK inhibits TGF-β1-induced airway smooth muscle cells proliferation and its potential mechanisms. Sci. Rep..

[157] Ding M., Xie Y., Wagner R.J., Jin Y., Carrao A.C., Liu L.S., Guzman A.K., Powell R.J., Hwa J., Rzucidlo E.M. (2011). Adiponectin induces vascular smooth muscle cell differentiation via repression of mammalian target of rapamycin complex 1 and FoxO4. Arterioscler. Thromb. Vasc. Biol..

[158] Weng M., Raher M.J., Leyton P., Combs T.P., Scherer P.E., Bloch K.D., Medoff B.D. (2011). ADPN decreases pulmonary arterial remodeling in murine models of pulmonary hypertension. Am. J. Respir. Cell Mol. Biol..

[159] Jiang T., Li Z., Zhao D., Hui B., Zheng Z. (2021). SOX18 enhances the proliferation and migration of airway smooth muscle cells induced by tumor necrosis factor-α via the regulation of Notch1 signaling. Int. Immunopharmacol..

[160] Li X., Zou F., Lu Y., Fan X., Wu Y., Feng X., Sun X., Liu Y. (2020). Notch1 contributes to TNF-α-induced proliferation and migration of airway smooth muscle cells through regulation of the Hes1/PTEN axis. Int. Immunopharmacol..

[161] Wen X., Yan J., Han X.R., Zheng G.H., Tang R., Liu L.F., Wu D.M., Lu J., Zheng Y.L. (2018). PTEN gene silencing contributes to airway remodeling and induces airway smooth muscle cell proliferation in mice with allergic asthma. J. Thorac Dis..

[162] Evans C.M., Raclawska D.S., Ttofali F., Liptzin D.R., Fletcher A.A., Harper D.N., McGing M.A., McElwee M.M., Williams O.W., Sanchez E. (2015). The polymeric mucin Muc5ac is required for allergic airway hyperreactivity. Nat. Commun..

[163] Zhu X., Cui J., Yi L., Qin J., Tulake W., Teng F., Tang W., Wei Y., Dong J. (2020). The Role of T Cells and Macrophages in Asthma Pathogenesis: A New Perspective on Mutual Crosstalk. Mediators Inflamm..

[164] Douwes J., Gibson P., Pekkanen J., Pearce N. (2002). Non-eosinophilic asthma: Importance and possible mechanisms. Thorax.

[165] Sze E., Bhalla A., Nair P. (2020). Mechanisms and therapeutic strategies for non-T2 asthma. Allergy.

[166] Draijer C., Peters-Golden M. (2017). Alveolar Macrophages in Allergic Asthma: The Forgotten Cell Awakes. Curr. Allergy Asthma Rep..

[167] van der Veen T.A., de Groot L.E.S., Melgert B.N. (2020). The different faces of the macrophage in asthma. Curr. Opin. Pulmon. Med..

[168] Murray P.J., Allen J.E., Biswas S.K., Fisher E.A., Gilroy D.W., Goerdt S., Gordon S., Hamilton J.A., Ivashkiv L.B., Lawrence T. (2014). Macrophage activation and polarization: Nomenclature and experimental guidelines. Immunity.

[169] Zhu L., Chen X., Chong L., Kong L., Wen S., Zhang H., Zhang W., Li C. (2019). Adiponectin alleviates exacerbation of airway inflammation and oxidative stress in obesity-related asthma mice partly through AMPK signaling pathway. Int. Immunopharmacol..

[170] Jung M.Y., Kim H.S., Hong H.J., Youn B.S., Kim T.S. (2012). ADPN induces dendritic cell activation via PLCγ/JNK/NF-κB pathways, leading to Th1 and Th17 polarization. J. Immunol..

[171] Tsang J.Y., Li D., Ho D., Peng J., Xu A., Lamb J., Chen Y., Tam P.K. (2011). Novel immunomodulatory effects of ADPN on dendritic cell functions. Int. Immunopharmacol..

[172] Wang L., Luo Y., Luo L., Wu D., Ding X., Zheng H., Wu H., Liu B., Yang X., Silva F. (2021). ADPN restrains ILC2 activation by AMPK-mediated feedback inhibition of IL-33 signaling. J. Exp. Med..

[173] Amarsaikhan N., Stolz D.J., Wilcox A., Sands E.M., Tsoggerel A., Gravely H., Templeton S.P. (2019). Reciprocal Inhibition of ADPN and Innate Lung Immune Responses to Chitin and Aspergillus fumigatus. Front. Immunol..

[174] Surendar J., Frohberger S.J., Karunakaran I., Schmitt V., Stamminger W., Neumann A.L., Wilhelm C., Hoerauf A., Hübner M.P. (2019). ADPN Limits IFN-γ and IL-17 Producing CD4 T Cells in Obesity by Restraining Cell Intrinsic Glycolysis. Front. Immunol..

[175] Wilk S., Scheibenbogen C., Bauer S., Jenke A., Rother M., Guerreiro M., Kudernatsch R., Goerner N., Poller W., Elligsen-Merkel D. (2011). Adiponectin is a negative regulator of antigen-activated T cells. Eur. J. Immunol..

[176] Shore S.A., Terry R.D., Flynt L., Xu A., Hug C. (2006). ADPN attenuates allergen-induced airway inflammation and hyperresponsiveness in mice. J. Allergy Clin. Immunol..

[177] Ramos-Ramírez P., Malmhäll C., Johansson K., Adner M., Lötvall J., Bossios A. (2020). Lung Regulatory T Cells Express ADPN Receptor 1: Modulation by Obesity and Airway Allergic Inflammation. Int. J. Mol. Sci..

[178] Helou D.G., Shafiei-Jahani P., Lo R., Howard E., Hurrell B.P., Galle-Treger L., Painter J.D., Lewis G., Soroosh P., Sharpe A.H. (2020). PD-1 pathway regulates ILC2 metabolism and PD-1 agonist treatment ameliorates airway hyperreactivity. Nat. Commun..

[179] Singh A.K., Stock P., Akbari O. (2011). Role of PD-L1 and PD-L2 in allergic diseases and asthma. Allergy.

[180] Nasiri Kalmarzi R., Fattahi N., Kaviani Z., Ataee P., Mansouri M., Moradi G., Yousefzade A., Abbassi J.M. (2017). Inverse correlation of soluble programmed cell death-1 ligand-1 (sPD-L1) with eosinophil count and clinical severity in allergic rhinitis patients. Allergol. Int..

[181] Yang Y., Li X., An X., Zhang L., Li X., Wang L., Zhu G. (2020). Continuous exposure of PM2.5 exacerbates ovalbumin-induced asthma in mouse lung via a JAK-STAT6 signaling pathway. Adv. Clin. Exp. Med..

[182] Steinke J.W., Lawrence M.G., Teague W.G., Braciale T.J., Patrie J.T., Borish L. (2021). Bronchoalveolar lavage cytokine patterns in children with severe neutrophilic and paucigranulocytic asthma. J. Allergy Clin. Immunol..

[183] Doherty T.A., Broide D.H. (2019). Airway innate lymphoid cells in the induction and regulation of allergy. Allergol. Int..

[184] Schuijs M.J., Png S., Richard A.C., Tsyben A., Hamm G., Stockis J., Garcia C., Pinaud S., Nicholls A., Ros X.R. (2020). ILC2-driven innate immune checkpoint mechanism antagonizes NK cell antimetastatic function in the lung. Nat. Immunol..

[185] Stier M.T., Zhang J., Goleniewska K., Cephus J.Y., Rusznak M., Wu L., Van Kaer L., Zhou B., Newcomb D.C., Peebles R.S. (2018). IL-33 promotes the egress of group 2 innate lymphoid cells from the bone marrow. J. Exp. Med..

[186] Helfrich S., Mindt B.C., Fritz J.H., Duerr C.U. (2019). Group 2 Innate Lymphoid Cells in Respiratory Allergic Inflammation. Front. Immunol..

[187] Wurtz O., Bajénoff M., Guerder S. (2004). IL-4-mediated inhibition of IFN-gamma production by CD4+ T cells proceeds by several developmentally regulated mechanisms. Int. Immunol..

[188] Hasegawa T., Okazawa T., Uga H., Kurata H., Mori A. (2019). Serum CXCL9 as a potential marker of Type 1 inflammation in the context of eosinophilic asthma. Allergy.

[189] Bocek P., Foucras G., Paul W.E. (2004). Interferon gamma enhances both in vitro and in vivo priming of CD4+ T cells for IL-4 production. J. Exp. Med..

[190] Chung S., Lee T.J., Reader B.F., Kim J.Y., Lee Y.G., Park G.Y., Karpurapu M., Ballinger M.N., Qian F., Rusu L. (2016). FoxO1 regulates allergic asthmatic inflammation through regulating polarization of the macrophage inflammatory phenotype. Oncotarget.

[191] Yamamoto R., Ueki S., Moritoki Y., Kobayashi Y., Oyamada H., Konno Y., Tamaki M., Itoga M., Takeda M., Ito W. (2013). ADPN attenuates human eosinophil adhesion and chemotaxis: Implications in allergic inflammation. J. Asthma..

[192] Weng M., Baron D.M., Bloch K.D., Luster A.D., Lee J.J., Medoff B.D. (2011). Eosinophils are necessary for pulmonary arterial remodeling in a mouse model of eosinophilic inflammation-induced pulmonary hypertension. Am. J. Physiol. Lung Cell Mol. Physiol..

[193] Martín-Orozco E., Norte-Muñoz M., Martínez-García J. (2017). Regulatory T Cells in Allergy and Asthma. Front. Pediatr..

[194] Baru A.M., Hartl A., Lahl K., Krishnaswamy J.K., Fehrenbach H., Yildirim A.O., Garn H., Renz H., Behrens G.M., Sparwasser T. (2010). Selective depletion of Foxp3+ Treg during sensitization phase aggravates experimental allergic airway inflammation. Eur. J. Immunol..

[195] Kearley J., Barker J.E., Robinson D.S., Lloyd C.M. (2005). Resolution of airway inflammation and hyperreactivity after in vivo transfer of CD4+CD25+ regulatory T cells is interleukin 10 dependent. J. Exp. Med..

[196] Tortola L., Pawelski H., Sonar S.S., Ampenberger F., Kurrer M., Kopf M. (2019). IL-21 promotes allergic airway inflammation by driving apoptosis of FoxP3+ regulatory T cells. J. Allergy Clin. Immunol..

[197] Sivakoti K., Chaya S.K., Jayaraj B.S., Lokesh K.S., Veerapaneni V.V., Madhunapantula S., Mahesh P. (2018). Evaluation of inflammatory markers MMP-2 and TIMP-1 in Asthma. Eur. Respir. J..

[198] Sands M.F., Ohtake P.J., Mahajan S.D., Takyar S.S., Aalinkeel R., Fang Y.V., Blume J.W., Mullan B.A., Sykes D.E., Lachina S. (2009). Tissue inhibitor of metalloproteinase-1 modulates allergic lung inflammation in murine asthma. Clin. Immunol..

[199] Procaccini C., Carbone F., Di Silvestre D., Brambilla F., De Rosa V., Galgani M., Faicchia D., Marone G., Tramontano D., Corona M. (2016). The Proteomic Landscape of Human Ex Vivo Regulatory and Conventional T Cells Reveals Specific Metabolic Requirements. Immunity.

[200] Hough K.P., Curtiss M.L., Blain T.J., Rui-Ming L., Trevor J., Deshane J.S., Thannickal V.J. (2020). Airway Remodeling in Asthma. Front. Med..

[201] Maslov L.N., Naryzhnaya N.V., Boshchenko A.A., Popov S.V., Ivanov V.V., Oeltgen P.R. (2018). Is oxidative stress of adipocytes a cause or a consequence of the metabolic syndrome?. J. Clin. Transl. Endocrinol..

[202] Cheng M., Liu H., Zhang T., Xu J. (2016). Different forms of ADPN reduce the apoptotic and damaging effect of cigarette smoke extract on human bronchial epithelial cells. Exp. Ther. Med..

[203] Grzela K., Litwiniuk M., Zagorska W., Grzela T. (2016). Airway Remodeling in Chronic Obstructive Pulmonary Disease and Asthma: The Role of Matrix Metalloproteinase-9. Arch. Immunol. Ther. Exp. (Warsz).

[204] Singh S.R., Sutcliffe A., Kaur D., Gupta S., Desai D., Saunders R., Brightling C.E. (2014). CCL2 release by airway smooth muscle is increased in asthma and promotes fibrocyte migration. Allergy.

[205] Ionescu L.I., Alphonse R.S., Arizmendi N., Morgan B., Abel M., Eaton F., Duszyk M., Vliagoftis H., Aprahamian T.R., Walsh K. (2012). Airway delivery of soluble factors from plastic-adherent bone marrow cells prevents murine asthma. Am. J. Respir. Cell Molec. Biol..

[206] Fang F., Liu L., Yang Y., Tamaki Z., Wei J., Marangoni R.G., Bhattacharyya S., Summer R.S., Ye B., Varga J. (2012). The adipokine ADPN has potent anti-fibrotic effects mediated via adenosine monophosphate-activated protein kinase: Novel target for fibrosis therapy. Arthritis Res. Ther..

[207] Park P.H., Sanz-Garcia C., Nagy L.E. (2015). ADPN as an anti-fibrotic and anti-inflammatory adipokine in the liver. Curr. Pathobiol. Rep..

[208] Liu Y., Xie L., Yang M., Tan X., Zeng Y., Zheng G., Chen Y., Chen P. (2017). PPAR-α improves the recovery of lung function following acute respiratory distress syndrome by suppressing the level of TGF-β1. Mol. Med. Rep..

[209] Enomoto N., Oyama Y., Yasui H., Karayama M., Hozumi H., Suzuki Y., Kono M., Furuhashi K., Fujisawa T., Inui N. (2019). Analysis of serum ADPN and leptin in patients with acute exacerbation of idiopathic pulmonary fibrosis. Sci. Rep..

[210] Paw M., Wnuk D., Kądziołka D., Sęk A., Lasota S., Czyż J., Madeja Z., Michalik M. (2018). Fenofibrate Reduces the Asthma-Related Fibroblast-To-Myofibroblast Transition by TGF-Β/Smad2/3 Signaling Attenuation and Connexin 43-Dependent Phenotype Destabilization. Int. J. Mol. Sci..

[211] Cui J., Lv Z., Teng F., Yi L., Tang W., Wang W., Tulake W., Qin J., Zhu X., Wei Y. (2021). RNA-Seq Expression Analysis of Chronic Asthmatic Mice with Bu-Shen-Yi-Qi Formula Treatment and Prediction of Regulated Gene Targets of Anti-Airway Remodeling. Evidence-Based Complem. Altern. Med..

[212] Green C.E., Turner A.M. (2017). The role of the endothelium in asthma and chronic obstructive pulmonary disease (COPD). Respir. Res..

[213] Shen L., Miao J., Yuan F., Zhao Y., Tang Y., Wang Y., Zhao Y., Yang G.-Y. (2013). Overexpression of ADPN promotes focal angiogenesis in the mouse brain following middle cerebral artery occlusion. Gene Ther..

[214] Mahadev K., Wu X., Donnelly S., Ouedraogo R., Eckhart A.D., Goldstein B.J. (2008). ADPN inhibits vascular endothelial growth factor-induced migration of human coronary artery endothelial cells. Cardiovasc. Res..

[215] Asosingh K., Weiss K., Queisser K., Wanner N., Yin M., Aronica M., Erzurum S. (2018). Endothelial cells in the innate response to allergens and initiation of atopic asthma. J. Clin. Investig..

[216] Tanaka Y., Kita S., Nishizawa H., Fukuda S., Fujishima Y., Obata Y., Nagao H., Masuda S., Nakamura Y., Shimizu Y. (2019). ADPN promotes muscle regeneration through binding to T-cadherin. Sci. Rep..

[217] Ito R., Higa M., Goto A., Aoshima M., Ikuta A., Ohashi K., Yokoyama S., Ohno Y., Egawa T., Miyata H. (2018). Activation of ADPN receptors has negative impact on muscle mass in C2C12 myotubes and fast-type mouse skeletal muscle. PLoS ONE.

[218] Matos Salame F., Fontoura Moreira M.A., Tesser L. (2015). Respiratory muscle strength evaluation in adults with asthma through maximal respiratory pressures measurements. Eur. Respir. J..

[219] Ather J.L., Van Der Vliet K.E., Mank M.M., Reed L.F., Dixon A.E., Poynter M.E. (2021). Obese adipose tissue modulates proinflammatory responses of mouse airway epithelial cells. Am. J. Physiol. Regul. Integr. Comp. Physiol..

[220] Sood A., Cui X., Qualls C., Beckett W.S., Gross M.D., Steffes M.W., Smith L.J., Jacobs D.R. (2008). Association between asthma and serum ADPN concentration in women. Thorax.

[221] Koksal B.T., Ozbek O.Y., Bayraktar N., Kinik S.T., Yazici A.C. (2015). Evaluation of Serum Leptin and ADPN Levels in Obese and Lean Asthmatic Children. Pediatr. Allergy Immunol. Pulmonol..

[222] Juel C.T., Ali Z., Nilas L., Ulrik C.S. (2012). Asthma and obesity: Does weight loss improve asthma control? A systematic review. J. Asthma Allergy.

[223] Bates J.H.T., Poynter M.E., Frodella C.M., Peters U., Dixon A.E., Suratt B.T. (2017). Pathophysiology to Phenotype in the Asthma of Obesity. Ann. Am. Thorac Soc..

[224] Vezir E., Civelek E., Dibek Misirlioglu E., Toyran M., Capanoglu M., Karakus E., Kahraman T., Ozguner M., Demirel F., Gursel I. (2021). Effects of Obesity on Airway and Systemic Inflammation in Asthmatic Children. Int. Arch. Allergy Immunol..

[225] Santos Coelho R., Castro Melo P.A., Dos Santos Silva H., De Cassia Ribeiro Silva R., Alvim Matos M.S., Lima Barreto M., Alcântara-Neves M.N., Viana de Figueiredo A.C., do Santos Costa R. (2021). ADIPOQ and LEP variants on asthma and atopy: Genetic association modified by overweight. Gene.

[226] Ramya K., Ayyappa K.A., Ghosh S., Mohan V., Radha V. (2013). Genetic association of ADIPOQ gene variants with type 2 diabetes, obesity and serum ADPN levels in south Indian population. Gene.

[227] Fenger R.V., Linneberg A., Vidal C., Vizcaino L., Husemoen L.L., Aadahl M., Gonzalez-Quintela A. (2012). Determinants of serum tryptase in a general population: The relationship of serum tryptase to obesity and asthma. Int. Arch. Allergy Immunol..

[228] Farzan S. (2013). The Asthma Phenotype in the Obese: Distinct or Otherwise?. J. Allergy.

[229] Götz A.A., Rozman J., Rödel H.G., Fuchs H., Gailus-Durner V., de Angelis M.H., Klingenspor M., Stoeger T. (2011). Comparison of particle-exposure triggered pulmonary and systemic inflammation in mice fed with three different diets. Part. Fibre Toxicol..

[230] Dixon A.E., Johnson S.E., Griffes L.V., Raymond D.M., Ramdeo R., Soloveichik A., Suratt B.T., Cohen R.I. (2011). Relationship of adipokines with immune response and lung function in obese asthmatic and non-asthmatic women. J. Asthma.

[231] Diaz J., Warren L., Helfner L., Xue X., Chatterjee P.K., Gupta M., Solanki M.H., Esposito M., Bonagura V., Metz C.N. (2015). Obesity shifts house dust mite-induced airway cellular infiltration from eosinophils to macrophages: Effects of glucocorticoid treatment. Immunol. Res..

[232] Ryu S.L., Shim J.W., Kim D.S., Jung H.L., Park M.S., Park S.H., Lee J., Lee W.Y., Shim J.Y. (2013). Expression of peroxisome proliferator-activated receptor (PPAR)-α and PPAR-γ in the lung tissue of obese mice and the effect of rosiglitazone on proinflammatory cytokine expressions in the lung tissue. Korean J. Pediatr..

